# Supplementary material for: Study protocol for economic evaluation of probiotic intervention for prevention of neonatal sepsis in 0–2-month old low-birth weight infants in India: the ProSPoNS trial
Source: BMJ Open. 2023 Mar 29;13(3):e068215. doi: 10.1136/bmjopen-2022-068215 (PMC10069556; doi:10.1136/bmjopen-2022-068215)
Supplement: Author's manuscript [file bmjopen-2022-068215.draft_revisions.pdf]

BMJ Open is committed to open peer review. As part of this commitment we make the peer review history of every article we publish publicly available.

When an article is published we post the peer reviewers' comments and the authors' responses online. We also post the versions of the paper that were used during peer review. These are the versions that the peer review comments apply to.

The versions of the paper that follow are the versions that were submitted during the peer review process. They are not the versions of record or the final published versions. They should not be cited or distributed as the published version of this manuscript.

BMJ Open is an open access journal and the full, final, typeset and author-corrected version of record of the manuscript is available on our site with no access controls, subscription charges or pay-per-view fees (http://bmjopen.bmj.com).

If you have any questions on BMJ Open's open peer review process please email info.bmjopen@bmj.com

A Study Protocol for Economic Evaluation of Probiotic Intervention for Prevention of Neonatal Sepsis In 0-2 Month Old Low Birth Weight Infants In India - The ProSPoNS Trial

Journal:	*BMJ Open*
Manuscript ID	bmjopen-2022-068215
Article Type:	Protocol
Date Submitted by the Author:	10-Sep-2022

Complete List of Authors:	sinha, anju; Indian Council of Medical Research, Reproductive and Child Health<br>Bahuguna, Pankaj; Post Graduate Institute of Medical Education and Research School of Public Health, Department of Community Medicine and School of Public Health<br>Gupta, Subodh; MGIMS, Sewagram, Community Medicine; MGIMS, Sewagram, Community Medicine<br>Kuruba, Yamini Priyanka; Indian Council of Medical Research, RBMCH<br>Poluru, Ramesh; The INCLEN Trust International,<br>Mathur, Apoorva; Indian Council of Medical Research, RBMCH<br>Raja, Dilip ; Indian Council of Medical Research, RBMCH<br>Raut, Abhishek; Mahatma Gandhi Institute of Medical Sciences, Department of Community Medicine<br>Mahajan, Kamaleshwar; Mahatma Gandhi Institute of Medical Sciences, Department of Community Medicine<br>Sudhakar, Rishikesh; INCLEN<br>Kulkarni, Bharati; National Institute of Nutrition, ;<br>Pandey, Ravindra Mohan ; All India Institute of Medical Sciences, Statistics<br>Arora, Narendra K. ; The INCLEN Trust International<br>Prinja, Shankar; Post Graduate Institute of Medical Education and Research, Community Medicine and School of Public Health; Post-graduate Institute of Medical Education and Research

Keywords:	Neonatal intensive & critical care < INTENSIVE & CRITICAL CARE, HEALTH ECONOMICS, Paediatric intensive & critical care < INTENSIVE & CRITICAL CARE

SCHOLARONE™ Manuscripts

A Study Protocol for Economic Evaluation of Probiotic Intervention for Prevention of Neonatal Sepsis In 0-2 Month Old Low Birth Weight Infants In India - The ProSPoNS Trial

Abstract

There is limited evidence on economic evaluations of interventions for prevention of neonatal sepsis. The ProSPoNS trial is a multicentre, double-blind, placebo-controlled trial to evaluate the role of probiotics in prevention of neonatal sepsis. The present protocol describes the data and methodology for the cost utility of the probiotic intervention alongside the controlled trial.

Methods: A societal perspective will be adopted in the economic evaluation. Direct medical and non-medical costs associated with neonatal sepsis and its treatment would be ascertained in both the intervention and the control arm. Intervention costs will be facilitated through primary data collection and program budgetary records. Treatment cost for neonatal sepsis and associated conditions will be accessed from Indian national costing database estimating healthcare system costs (NHSCD). A cost –utility design will be employed with outcome as incremental cost per disability-adjusted life year (DALY) averted. Considering a time-horizon of 6 months, trial estimates will be extrapolated to model the cost and consequences among high-risk neonatal population in India. A discount rate of 3% will be used. Impact of uncertainties present in analysis will be addressed through both deterministic and probabilistic sensitivity analysis. In addition, budget impact analysis and feasibility analysis will be undertaken to inform affordability and sustainability of the intervention if considered for implementation.

Expected outcomes: This study focuses on evaluating the cost-utility of probiotic intervention for prevention of neonatal sepsis. Findings from the cost-utility analysis will be crucial for induction of the intervention within the ambit of existing governmental programs, if found to be efficacious.

Ethics approval: Has been obtained from EC of each participating site as well as from the ERC of LSTM, UK.

Strengths: There is limited evidence on the value for money from prevention of neonatal sepsis. This study would add to knowledge regarding the economic implications of the probiotic intervention by extrapolating the costs and effectiveness to a wider population of newborn infants in the Low and Middle income countries (LMICs), and over a time horizon with help from modelling. Based on the results a widespread implementation strategy of the probiotic intervention may be planned by the policy makers.

For peer review only

Limitations: Assumptions made for long term implications from a relatively short duration trial may lead to limitation. Sensitivity analyses will be undertaken to address uncertainties introduced due to this assumption.

Introduction

There is progress towards attaining the 3rd Sustainable Development Goal (SDG) of under-five (U5) mortality of 25 per 1000 live births by 2030, in India. Neonatal mortality rate is the most important contributing factor of the U5 mortality [1, 2]. Further decline in neonatal mortality may require newer interventions for prevention or treatment of neonatal infections like meningitis, pneumonia, septicaemia that result in more than a quarter of the 1 million neonatal deaths every year in India [2]. The ProSPoNS trial [3] proposes to evaluate the role of probiotics in prevention of neonatal infections (sepsis) among the vulnerable group of low birth weight infants contributing the highest rates of morbidity and mortality. A larger sample size and precise outcome measures differentiate this trial from the earlier conducted pilot study that showcased a 21% non-significant decline in morbidity due to neonatal sepsis in the intervention arm. Considering the 30% prevalence of low-birth weight (LBW) in India and 30 % mortality due to neonatal sepsis, even a small decline in the incidence of sepsis would translate to a large number of new born lives saved. [3]

If the intervention is proven to be efficacious, it would be important to know whether it is also a cost effective intervention. There are few examples in the literature where economic evaluations have been conducted on other effective interventions to prevent neonatal sepsis.[4,5] It cannot be overemphasized that along with clinical effectiveness of interventions, an estimate of economic effectiveness either in terms of costs or utilities (DALYs and QALYs) is required to make correct choices about their inclusion in public health programs. This is of immense importance in presence of multiple competitive options where prioritization is needed.

Ranjeva et al. [6] have quantified the scope of the public health and economic burden of neonatal sepsis in Sub-Saharan Africa. However, evidence on the health economics aspect of neonatal sepsis prevention effect of probiotics has not been ascertained so far. We propose to evaluate the cost utility of probiotic intervention for prevention of neonatal sepsis within the ProSPoNS trial,

Background

Study Setting & Study population:

For peer review only

ProSPoNS trial is a Phase III multicentre randomized, double-blind placebo-controlled trial, being conducted at six study sites across India. It is a global Health trial funded by the UK Research and Innovation (UKRI).

The Study is ongoing at 6 sites across India: Jawaharlal Institute of Postgraduate Medical Education and Research (JIPMER), Puducherry, Mahatma Gandhi Institute of Medical Sciences (MGIMS), Wardha, King Edward Memorial Hospital (KEM Hospital), Pune, Asian Institute of Public Health (AIPH), Bhubaneswar (with recruitments from SCB Medical College and Hospital, Cuttack), Subharti Medical College (SMC) and Lala Lajpat Rai Memorial Medical (LLRM) College District women hospital, Meerut and Lady Hardinge Medical College (LHMC), Kalawati Saran Children's Hospital (KSCH), Delhi. The primary objective is to estimate a 30% reduction in the incidence of neonatal sepsis in the intervention arm with daily supplementation of 1ml (10 billion CFU/ml) of Vivomixx drops over a period of 30 days in 0-2-month-old LBW infants. 6144 Low birth weight (between 1500-2500 g) infants would be recruited to the study from the 6 study sites located in Bhubaneswar, Meerut, New Delhi, Puducherry, Pune, and Sewagram, between ages 3-7 days of life.

Theory of change

More than one-third of the estimated four million neonatal deaths around the world each year are caused by severe infections, and a quarter - around one million deaths - is due to neonatal sepsis/pneumonia alone. Panigrahi et al in their study which was conducted in the year 2017 reported that the Culture confirmed incidence of sepsis as 6.7/1000 births with 51% Gram negatives (Klebsiella predominating) and 26% Gram positives (mostly Staphylococcus aureus). The Sustainable Development Goal for child survival cannot be achieved without substantial reductions in infection-specific neonatal mortality (1). Due to non-specific signs and symptoms, sepsis is difficult to diagnose. The chances of recovery and survival depend on the correct diagnosis, referral, reaching a facility and adequate treatment by appropriately-trained health workers and high-quality services.

Low Birth Weight is one of the important indirect causes of deaths in neonates accounting for 40-80% of neonatal deaths worldwide (2). LBW neonates are immune compromised and manifest poor cognitive functions (7). Since LBW infants are more prone to infections, neonatal mortality and morbidity may be decreased by preventing neonatal infection.

Antibiotic therapy is the mainstay of current therapeutic management of neonatal infections. For serious infections in new borns and young infants, there is a lack of guidelines on rational antibiotic use (8). In the absence of rapid diagnostics, non-judicious use of antibiotics contributes to the rising

For peer review only

antimicrobial resistance. Antibiotic overuse is linked with disturbing the gut ecosystem. The problem of drug resistance outweighs the fast pace of newer generation antibiotic production.

Currently, there are no preventive interventions available for neonatal sepsis other than general measures of hand washing, exclusive breastfeeding, etc. However evaluating immunotherapy with immune globulin, probiotics, myeloid colony-stimulating factors, glutamine supplementation, recombinant human protein C and lactoferrin are being evaluated as adjuvants for the prevention of neonatal sepsis (9). Probiotics are non-pathogenic microbes which are capable of exerting positive health effects in the host body through various mechanisms (10) (11). It acts by suppressing the growth of potential pathogens and their epithelial attachment. It also works by secreting anti-microbial substances or by stimulating host expression of protective molecules. Besides, it can produce a barrier against the harmful pathogens at higher levels. It stimulates the process of immunosuppressive agent's production in the host which suppress the inflammatory responses or accelerate the immunologic mechanism that clears the infection. Probiotics stimulate normal microbe-microbe and host-microbe interactions positively, thereby enhance the protection provided by commensal flora through competitive interactions, pathogenic antagonism, and antimicrobial factors. We hypothesize that immunomodulation/ immunopotentiation with probiotics may prove to be an alternative for the prevention of neonatal sepsis.

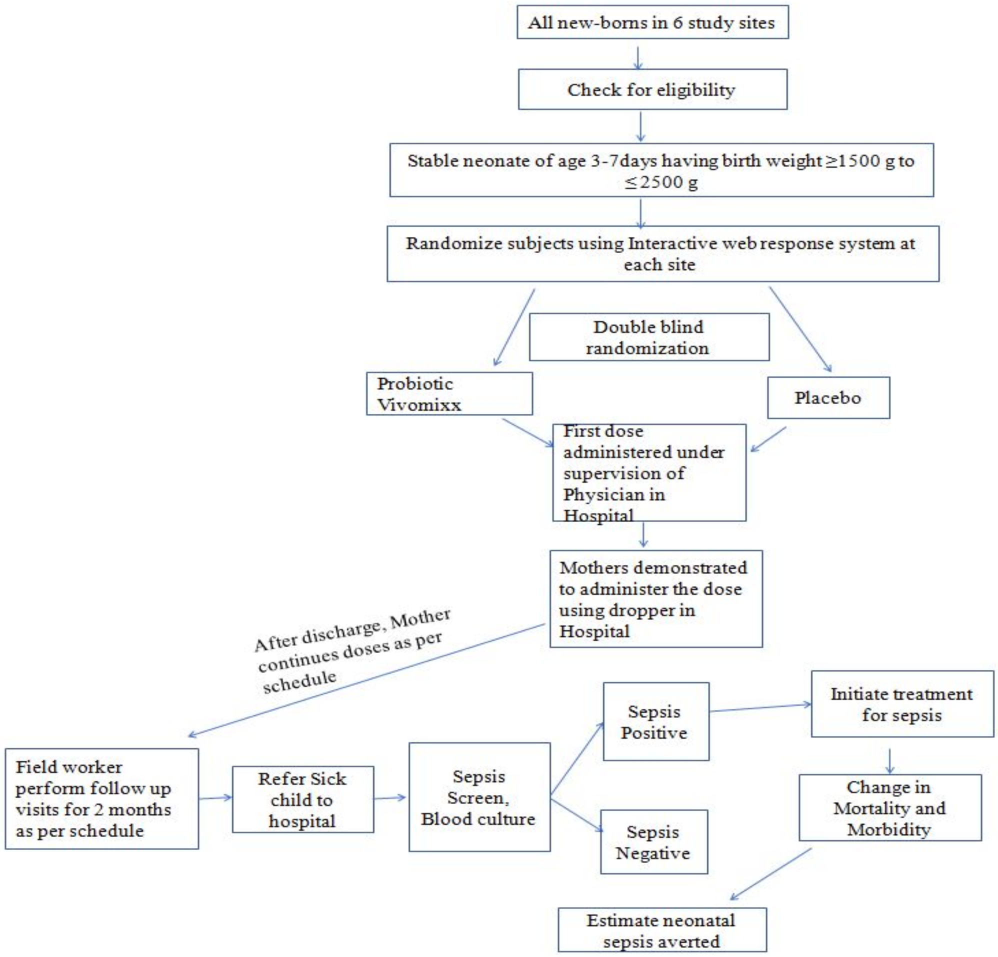

Fig.1 Conceptual framework to prevent neonatal sepsis using probiotics.

Objectives: To assess the cost utility of using probiotics as prophylaxis for sepsis among LBW neonates of age 0 to 2 months.

Study Methodology

A cost utility analysis is proposed to be conducted alongside the multicentre RCT. Randomized controlled trials (RCT) are considered the 'gold standard' as they produce a reliable the best evidence of treatment effect. The economic evaluation alongside RCT provides opportunity to collect data both on disease-specific disability and resource use.

Model Overview:

General Description:

This protocol describes the methodology for economic evaluation within a phase III RCT – the ProSPoNS trial. The details are described in the following sections.

Decision Model

In view of the shortcomings of treatment effect measure coming from a single RCT, we also propose using a model-based approach which will allow synthesizing information from all sources for a health technology enabling us to explore scenarios not explicitly found in the trial data. Moreover, the modelling approach would enable to estimate the long-term benefits of intervention incorporating quality of life measures. Furthermore, it allows accounting for uncertainties present in assumptions and data used for economic evaluation, which has implications for policy question under consideration. The clinical outcomes from trial would be sepsis episodes averted as an effect of probiotics use in the intervention arm. In order to assess the long-term effects, we propose to use 'Disability adjusted life years' (DALY) as the measure of health outcome which accounts for both disability and quantity of life. A decision-analytic model following a systematic approach in bringing together evidence on clinical effectiveness, health-related disability of life and costs associated with the probiotic intervention as compared to placebo will be used with the aim to provide information on whether the reduction in neonatal sepsis incidence justifies the incremental costs. **Figure 2** depict the decision model for the proposed study.

For peer review only

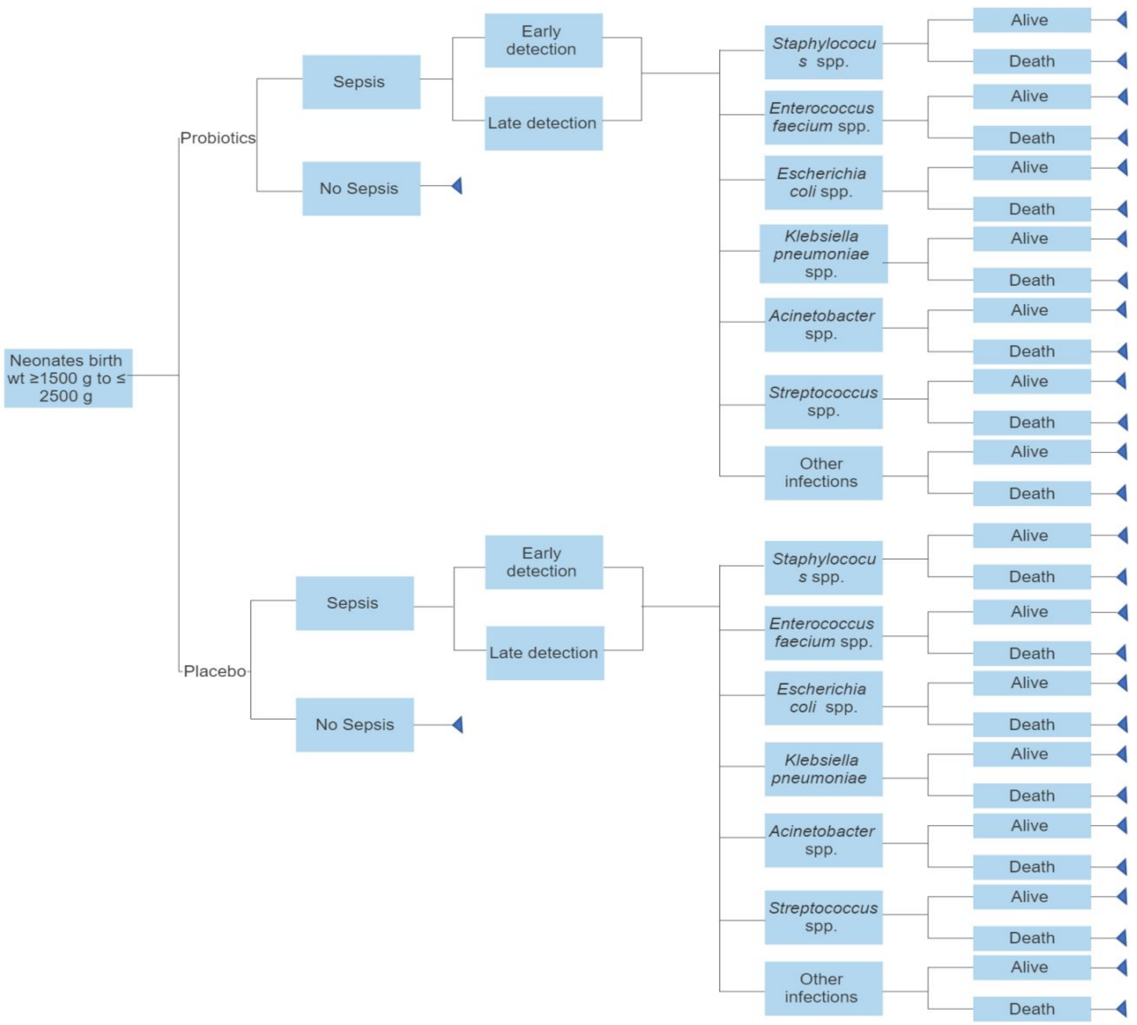

Fig.2. Decision tree for cost utility analysis of using probiotics as prophylaxis for sepsis among neonates aged 0 to 2 months.

Outcome measures: Primary outcome measure for economic evaluation (within-trial CUA) would be sepsis episodes and related deaths averted in the intervention arm as compared to the placebo arm, which translates to gain in life years and DALYs averted as long-term measure.

Data requirements

Table- 1: Data requirements for economic evaluation:

Data Type	Significance for the Model	Source of Data
Demographic	- Estimate the baseline parameters<br>- Estimate target population	Primary data collection; Census of India
Treatment effects & Incidence	- Efficacy and safety	RCT Database

of Adverse Events	parameters<br>- Cost estimation<br>- Outcome assessment	
Background Mortality data	- Estimate natural mortality in the target population	SRS, Published literature
Utilities	- Estimate DALY as long-term outcome measure	Global Burden of Diseases estimates (2011)
Resource use and price data	- Cost estimation	Primary data collection; NHSC-Prinja et al
Epidemiological data	- Estimate baseline risk of clinical events	Published literature; Primary data collection (Control arm of the RCT)

RCT- Randomised Control Trial, SRS- Sample Registration System, NHSCD- National Health Systems Costing Database

Utilities data would allow incorporating the relative impact of a health technology (i.e. probiotics) on the neonatal health outcomes in terms of disability burden. This will be estimated as DALYs averted. Utility data would be obtained from Global disease burden estimates 2017. Cost data are important drivers of cost-effectiveness, as they quantify the resource consumption associated with use of health technologies.

Decision-analytic model would be populated with cost data from the societal perspective: direct medical and non-medical cost borne by patients; and direct medical cost borne by the health care system. A national health system costing database (NHSCD) to estimate health care system's costs in India has been developed. [12] Data on patient's out-of-pocket (OOP) expenses would be collected during the conduct of the RCT as primary data. (Appendix: tool for OOP).

A health economic evaluation using cost- utility analysis (CUA) design will be conducted within the ProSPoNS trial. The results would provide the incremental costs and health gains of the probiotic intervention in absence of any prevention modality being implemented or available. Decision-analytical modelling using the trial results would be extrapolated to the target population and time horizon to inform policy on the potential for incremental costs offsets attributable to prevention of morbidity with the proposed intervention. The CUA will use the present results as cost per case of neonatal sepsis prevented. CUA will present the results as cost per disability-adjusted life year averted. Intervention costs and effects will be extrapolated to the Indian neonatal population. Modelling will be used for the target population, time horizon, and the decision context to provide

For peer review only

much required information for the policymakers. It would guide further decision for up scaling the intervention, if it is found to be efficacious. If the incremental costs of the intervention can be offset by the gains in terms of effectiveness (i.e. DALYs) it may be presented for induction within the ambit of existing governmental program. Modelled CUA results can be presented as health - adjusted life years saved and savings of the health care system by disease prevention. We will calculate the incremental cost-effectiveness ratios as the difference in the costs between the intervention and the comparator divided by the difference in health outcomes.

Effectiveness assessment:

Prevention of severe sepsis in the early neonates will prevent the long term morbidity like neurodevelopmental disability risk associated with sepsis (13) and reduce mortality due to pneumonia, meningitis, and neonatal sepsis and hospital admissions due to any infectious diseases (14).

We propose to use the short time horizon to assess the sepsis averted as a result of probiotics supplementation for one month and model the change in DALYs in the intervention group as a long-term measure.

Study Trial

Total of 6144 (N) new-born babies with LBW would be screened for eligibility and randomly assigned to probiotic (n=3072) or placebo (n=3072) groups and administered 1 ml of the probiotic suspension daily for 30 days. The study would use IWRS for the purpose of stratified randomization by birth weight, sex and study site. Trained field workers will make home visits daily for first 7 days and thereafter thrice in a week till day-30 of the follow up period to ensure and monitor adherence to the intervention and collect morbidity information. After the 30-day intervention period, home visitation will continue at weekly interval till day-60 of life. During home visitation as per schedule trained fieldworkers would screen infants for symptoms/ signs of PSBI/ Clinical illness/ Clinical severe infection. To record the AE/SAE events (if any), continuous monitoring and reporting of ongoing medical conditions, signs symptoms will be conducted by field worker during their scheduled follow-up home visits using software on handheld device during the two-months period of follow up. Presence of any danger signs would be evaluated, and need for referral would be assessed: If a child is found to have any symptoms/ signs from the list, the field worker will accompany the infant to facility for examination by study physician (referral). The study physician would examine the infant again for symptoms/ signs of PSBI/ Clinical illness/ Clinical severe infection and confirm the diagnosis. Investigations such as blood culture, sepsis screen would be performed for suspected

For peer review only

cases of neonatal sepsis by study physician. If required the infant would be hospitalized for treatment. Data on outcomes, lab investigation and treatment during clinical management of the sick new-born will be collected and serious adverse events will be reported as per regulatory guidelines. Blood culture isolates will be shipped by site to microbiology lab at All India Institute of Medical Sciences (AIIMS), New Delhi for quality assurance purpose.

The study will be implemented according to MRC guidelines for management of global health trials and will be governed by a Trial Steering Committee and a Data Management Committee.

The primary outcomes: 1. **Sepsis:** defined as one or more clinical symptoms suggestive of sepsis with a microbial isolate on blood culture; or a neonate with sterile blood culture with at least 2 sepsis screen markers being abnormal (Absolute neutrophil count<1500/mm3; TLC<5000/mm3; Immature to total neutrophil ratio > 0.2; Immature to total neutrophil ratio > 0.2;CRP >1.2 mg/dl, ESR>15 mm).

2. **Possible Serious Bacterial Infection (PSBI):** One or more clinical signs like fever (temperature ≥ 38 °C), low body temperature (< 35.5 °C), not feeding well, convulsions, fast breathing (60 breaths per minute or more), severe chest in-drawing, movement only when stimulated or no movement at all in infants less than 7 days old confirmed by study physician.

Secondary outcomes: 1. Clinical severe infection: One or more clinical signs like severe chest in-drawing, not feeding well, fever (temperature ≥ 38 °C), the movement only when stimulated as confirmed by the study physician, low body temperature (< 35.5 °C).

2. Critical Illness: One or more of clinical signs like unable to feed at all, unable to cry, no movement on stimulation, bulging fontanelle, cyanosis as confirmed by the study physician and convulsions. The flow chart below (Figure 1) depicts the study design and the study processes that will be followed during the implementation of the proposed research work (Diagram, to be finalised after finalisation of methods). Primary and secondary outcomes would be compared between the two study arms to assess clinical effectiveness of probiotic intervention.

Costing: In the current study, cost of Vivomix (intervention), cost of routine home based post natal care (HBPNC), cost of illness management, drug costs, vitamin/mineral supplement costs, laboratory investigation charges, and doctor consultation, bed charges are categorized as direct medical costs and the informal payments and travel are categorized as the direct non-medical costs. Loss of wages of parents and other caregivers due to absenteeism from work and is categorized as indirect costs. All costs related data would be collected from all six study sites in a representative manner.

For peer review only

Sample size (Costing):

We calculated the sample size to estimate the mean out-of-pocket (OOP) expenditures among the neonates with illness (here PSBI). A power of 80% and 5% level of significance was assumed for sample size calculation. The baseline estimate for OOP among neonates with PSBI was accessed from an Indian study. (1) Assuming a mean OOP of □2003 (Std deviation: □3517) for neonates with PSBI and an error margin of 7.5%, the minimum sample size computed to estimate the OOP was 1998. In view of high variability in the OOP data, the error margin of 7.5% was considered appropriate for sample size calculation. The data on OOP will be captured for a sub-sample i.e. 1998 neonates, out of total sample recruited in the study. At each of the six subjects recruitment sites data would be collected on 333 subjects for this sub-study. The OOP data will be collected irrespective of status of neonates belonging to two study arms. The formula used for sample size calculation is given below:

$$n = \frac{Z^2_{1-a/2}\, s^2}{d^2}$$

Where; n= minimum estimated sample size
Z^2_{1-a}= table value from standard normal distribution
s^2= standard deviation of outcome variable in study population
d^{2} = error margin (or absolute precision)

Data collection & Estimation of costs: Data collection for the current study would be completed in one year duration from September 2022 to August 2023 (to cover any variation with seasonality). Data on out-of-pocket expenditures (OOP) will be collected on a sub-sample of 333 subjects drawn from the total trial population in a representative manner.

Socio- demographic data on education, occupation of parents of enrolled children is being collected on study forms. Parents would be asked about any illness during the study period and any expenditures on account of care seeking, visits to facilities, hospitalization and treatment. Direct medical and non-medical costs due to neonatal sepsis would be ascertained in all 333 subjects at the six study sites. Data on OOP expenditure in public or private health facilities would be elicited.

Intervention cost would be facilitated through primary data collection and the program budgetary records. The cost of treatment for neonatal sepsis and related diseases at secondary and tertiary level will be ascertained from the NHSCD[15,16] and published literature in Indian context.[12] These sources provides estimates on cost per in-patient (IP) admission, cost of intensive care unit (ICU)

For peer review only

admission, cost per out-patient (OP) consultation accounting for resources consumed for service provision such as medical (drugs, diagnostics, procedural, hospital charges) and non-medical (transportation, boarding & lodging). In addition, cost related to IEC material, monitoring & supervision and other administrative costs will also be estimated.

Table 2: Key methods and assumptions for economic evaluation:

**Component**	**Consideration/Approach**	**Justification**
Intervention	- Vivomixx (8 strain probiotics) + Sepsis management<br>- Dose: 1ml/day (10 billion CFU/ml) for 30 days<br>- Intervention delivery: Under the supervision of study staff (field worker)	Effectiveness of Vivomixx for preventing neonatal sepsis is being assessed under trial
Comparator	- Placebo	- Effectiveness of Vivomixx is being assessed under trial against Placebo<br>- Use of antibiotic is routine practice for sepsis management.
Target Population	- High-risk LBW neonates (Both pre-term and small for-date babies)	Most vulnerable high-risk population with high morbidity & mortality
Objective	- To assess the incremental cost per DALY averted with use of probiotics for prevention of neonatal sepsis compared the placebo	Besides effectiveness, evidence on cost-effectiveness is mandatory for adoption of treatment/intervention under public policy
Target Audience/Policy Use	- National Health Mission (NHM)<br>- Any other	This intervention is to be implemented under RMNCH program
Method	- Both model and trial based	- Short –term outcome estimates will be used for trial<br>-Use of model-based CEA will enable us to assess long-term outcomes (and sub-group analysis)
Perspective	- Societal perspective	OOP burden associated with sepsis management among LBW neonates
Analytic Horizon	- 6 months	To model long-term outcomes (DALYs)
Costs	- Health system costs (Vivomixx	Both health system and patients' costs as per societal perspective

For peer review only

	cost; Cost of routine HBPNC care; cost of illness management etc.)<br>- OOP expenditures (Doctor's consultation; bed charges; Drug costs; Vitamin/Mineral supplement costs; Investigations; Procedures; Informal payments; Travel cost etc.	considered for CEA
Outcomes	- Sepsis episodes and deaths averted<br>- DALYs<br>- Hospitalization averted<br>- Life years gained	In line with study design (cost-effectiveness/cost-utility analysis)
Discount rate	- 3%	As per Indian reference case

Total cost will be comprised of cost of intervention, cost of management of sepsis and associated complications and patient costs as OOP. Similarly, total cost in the control arm will be estimated in the absence of intervention. Annual discount rate of 3% will be used wherever applicable. In the present study both model based approach as well as trial data would be used. Data management and analysis will be done using MS-Excel.

Other Analyses: Uncertainty analyses, Budget impact analysis, Stakeholder analysis would be included.

Uncertainty Analysis: Both deterministic and probabilistic sensitivity analysis (PSA) will be undertaken to address the effect of uncertainties present in analysis. For PSA, all the parameter values will be varied within a plausible range. Based on nature of parameter, an appropriate probabilistic distribution will be assigned such as beta distribution for risk and utility parameters, gamma distribution for cost parameters, uniform distribution for demographic parameters etc. The results under PSA will be simulated 999 times to report a 95% confidence interval for ICERs.

Budget Impact Analysis

A Budget impact analysis is proposed to be conducted to estimate the financial consequences of increasing uptake of probiotic supplementation for LBW infants in the neonatal care under NHM, over the near future (3 to 5 years). Data regarding the target population of LBW infants will be

For peer review only

available by extrapolating the data from the RCT, as will be other epidemiological inputs to populate the budget-impact model. At present there are no alternative prevention modalities for neonatal sepsis present (other than hand hygiene and exclusive breastfeeding). Therefore, assumption would be made regarding the uptake of probiotic supplementation (based on the results of the study).

Stakeholder analysis: A range of potential stakeholders will be consulted, involving experts such as neonatologists/paediatricians, academics, policy makers, program managers, industry, patient group representatives etc. Multiple consultations with stakeholders will be done during the conduct of study as well as dissemination of study results.

Patient and Public Involvement: Patients or caregivers or members of public were not involved in the development of this protocol

Discussion:

The ProSPoNS trial is evaluating the role of probiotics intervention in prevention of neonatal infections, a major cause for neonatal mortality. To date no prevention modalities other than hand hygiene and exclusive breastfeeding have been successfully incorporated in programs for prevention of neonatal infections. Panigrahi el al (2017) established benefit of Lactobacillus planitarum with fructooligosaccharides intervention in prevention of sepsis and related deaths in new-borns; however, the study did not assess the cost effectiveness of the intervention. There is no study in the literature evaluating the cost-effectiveness of this novel disease prevention technology. We have proposed such evaluation alongside the ProSPoNS trial.

A RCT design, considered a gold standard for such evaluations is being used in the trial. However, there are disadvantages of using RCT data quoted in the literature. Data from a single RCT may not be adequate for estimation of cost utility as RCTs mainly focus on a single intervention and have small follow up period, thus, lack to provide evidence on effectiveness of multiple alternatives and situations beyond the trial conditions. The treatment effect measured in a single study may be relevant for a particular setting but has limited generalizability of results in populations other than the one studied. [17, 18, 19]. We propose using a modelling approach to overcome the above mentioned shortcomings of RCT data.

There are a few examples of estimation of cost-effectiveness of disease preventive technologies in the neonatal population. A study conducted in Sub-Saharan Africa estimated that neonatal sepsis resulted in 5.29-8.73 DALYs lost annually accounting for an economic burden from $10 billion to $469 billion (per annum). It was recommended that a strategic plan for the treatment and prevention

For peer review only

of neonatal sepsis may reduce this burden.[6] A cost-effective analysis conducted in Ghana reported that alcohol-based hand rub (ABH) effectively reduced the patient cost of neonatal bloodstream infection (BSI), Hospital cost associated with BSI, length of hospital stay, and deaths due to BSI by 41.7%, 48.5%, 50% and 73% respectively. It was also estimated that ABH reduced the incidence of hospital-acquired neonatal infections by 16%.[4]. Current study would estimate the cost-effectiveness of a novel neonatal sepsis prevention modality.

In considering cost-effectiveness, measures on treatment effect over longer time horizon than RCTs are often required. Therefore we have proposed a modelling approach in addition to the use of primary data coming from the ongoing RCT. This approach would use and provide a wider range of assumptions/parameters from multiple data sources and evaluate the long term utility of the probiotic intervention in disease prevention.

Expected outcomes of study and policy implications: The health economics evaluation would generate data on cost involved with the new intervention as compared to the control arm with uncertainty range, Incremental costs, and health outcomes for both study arms with uncertainty range, ICER, sensitivity and threshold analyses. It would be helpful for the policy maker to understand the cost utility of the probiotic intervention in presence of other competing priorities.

Strengths: There is limited evidence on the value for money from prevention of neonatal sepsis. This study would add to knowledge regarding the economic implications of the probiotic intervention by extrapolating the costs and effectiveness to a wider population of newborn infants in the Low and Middle income countries (LMICs), and over a time horizon with help from modelling. Based on the results a widespread implementation strategy of the probiotic intervention may be planned by the policy makers.

Challenges: Assumptions made for long term implications from a relatively short duration trial may lead to limitation. Sensitivity analyses will be undertaken to address uncertainties introduced due to this assumption.

Registration: The protocol has been approved by the regulatory authority (Central Drugs Standards Control Organisation; CDSCO) in India (CT-NOC No. CT/NOC/17/2019 dated 01 March 2019). The ProSPoNS trial is registered at the Clinical Trial Registry of India (CTRI). CTRI/2019/05/019197. Registered on 16 May 2019

For peer review only

Ethics and Dissemination: Approval has been obtained from EC of each of the six participating site as well as from the ERC of LSTM, UK. A peer reviewed article will be published after completion of the study. Findings will be disseminated to in the community of the study sites, with academic bodies and policy makers.

Author affiliations: Anju Pradhan Sinha[1], Pankaj Bahuguna[2], Subodh S. Gupta[3], Kuruba Yamini Priyanka[1], Ramesh Poluru[4], Apoorva Mathur[1], Dilip Raja Koppula[1], Abhishek V. Raut[3], Kamlesh Mahajan[3], Rishikesh Sudhakar[4], Bharati Kulkarni[1], Ravindra Mohan Pandey[5], Narendra Kumar Arora[4], Shankar Prinja[2] & the ProSPoNS Trial group of investigators.

1. Indian Council of Medical Research, New Delhi
2. Post Graduate Institute of Medical Education and Research, Chandigarh
3. Mahatma Gandhi Institute of Medical Sciences, Wardha
4. INCLEN Trust International, New Delhi
5. All Indian Institute of Medical Science, New Delhi

Contributors: APS, SSG, NKA, AR, RP conceptualised the study. Pankaj Bahuguna, Bharti Kulkarni and Shankar Prinja contributed to study design, tools and analysis plan; ProSPoNS trial investigators will be involved in conduct of the study and data collection. All authors contributed to, reviewed, and refined the protocol and approved the final manuscript.

List of Authors: ProSPoNS Trial group of investigators Ashish Bavdekar, Sunil Sazawal, Sushma Nangia, Sailaja Nandan Parida, B. Adhsivam.

Funding: The study is funded by the Department of Health and Social Care, the Department for International Development, the Global Challenges Research Fund, the Medical Research Council, and Welcome Trust. The funding body has no role in the designing of the study and collection, analysis, and interpretation of data and in writing the manuscript.

Conflicts of Interests: The Authors declare that they have no conflicts of Interests.

Data Statement: As this is a study protocol at this point we can't provide data statement.

Acknowledgements: We would like to thank the Indian Council of Medical Research (ICMR) for the permission to conduct the study and the UK Research and Innovation, Medical Research Council, UK, for funding the trial under the Global Health Trials [JGHT] scheme 8, jointly funded by the DFID/NIHR/MRC/Wellcome Trust. We thank Dr. Soumya Swaminathan, Ex-Director General of the Indian Council of Medical Research (ICMR), New Delhi and late Prof. M.K. Bhan, for their

For peer review only

motivation, encouragement and support with this study. The authors also thank M/s Next Gen Pharma India Pvt. Ltd., India, for being the trial partners (supply of the Active Pharmaceutical Ingredient (API)) and M/s Cadila Healthcare Ltd., India, for agreeing to manufacture and supply the finished formulation: probiotic drops and corresponding placebo used in the study. The authors especially thank the Liverpool School of Tropical Medicine Research Ethics Committee (LSTM REC), IEC of each participating site (participant recruitment site and laboratory site), the Data Monitoring Committee, and the Trial Steering Committee (TSC) for their review and inputs.

References:

1. India Newborn Action Plan (INAP): National Health Mission [Internet]. [Cited 2021 Mar 10]. Available from: https://nhm.gov.in/index4.php?lang=1&level=0&linkid=153&lid=174 https://www.newbornwhocc.org/INAP_Final.pdf.
2. Sachs JD. From millennium development goals to sustainable development goals. Lancet. 2012; 379(9832):2206–11. https://doi.org/10.1016/S0140-673 6(12)60685-0.
3. Sinha AP, Gupta SS, Poluru R, Raut AV, Arora NK, Pandey RM, Sahu AR, Bethou A, Sazawal S, Parida S, Bavdekar A. Evaluating the efficacy of a multistrain probiotic supplementation for prevention of neonatal sepsis in 0–2-month-old low birth weight infants in India—the "ProSPoNS" Study protocol for a phase III, multicentre, randomized, double-blind, placebo-controlled trial. Trials. 2021 Dec; 22(1):1-2.
4. Fenny AP, Otieku E, Labi KA-K, Asante FA, Enemark U (2022) Cost-effectiveness analysis of alcohol handrub for the prevention of neonatal bloodstream infections: Evidence from HAI-Ghana study. PLoS ONE 17(3): e0264905.
5. Nice Clinical Guideline 2012 on Antibiotic for early-onset neonatal infection.
6. Ranjeva SL, Warf BC, Schiff SJ. Economic burden of neonatal sepsis in sub- Saharan Africa. BMJ Glob Health2018; **3:e000347**. doi:10.1136/ bmjgh-2017-000347
7. Allen L and Gillespie S. What works? A review of the efficacy and effectiveness of nutrition interventions. ACC/SCN Nutrition Policy Paper No 19, ADB Nutrition and Development series No 5, 2001: 5-22.
8. Lin HC, Su BH, Chen AC, et al. Oral probiotics reduce the incidence and severity of necrotizing enterocolitis in very low birth weight infants. Paediatrics 2005; 115(1): 1-4
9. Wolkowiez MC, Benjamin DK, Capparelli E. Immunotherapy in neonatal sepsis: Advances in treatment and prophylaxis. Curr Opin Pediatr 2009; 21(2):177-181.
10. MarteanP, Seksik P, Jian R Probiotics and intestinal health effects: a clinical perspective. Br J. Nutr;2002,Sep; 88 Suppl 1:S51-7

For peer review only

11. R. Balfour Sartor, Probiotic therapy of intestinal inflammation and infections. Current opinion in Gastroenterology 2005, 21:44-50
12. Prinja S, Brar S, Singh MP, et al. Process evaluation of health system costing - Experience from CHSI study in India. PLoS One. 2020; 15(5):e0232873. Published 2020 May 13. doi:10.1371/journal.pone.0232873
13. Cai S, Thompson DK, Anderson PJ, Yang JY. Short- and Long-Term Neurodevelopmental Outcomes of Very Preterm Infants with Neonatal Sepsis: A Systematic Review and Meta-Analysis. Children 2019, 6, 131; doi:10.3390/children6120131
14. Wiens MO, Kissoon N, Holsti L. Challenges in pediatric post-sepsis care in resource limited settings: a narrative review. Transl Pediatr 2021; 10(10):2666-2677.
15. Prinja S, Chauhan AS, Rajsekhar K, Downey L, Bahuguna P, Sachin O, Guinness L. Addressing the cost data gap for universal healthcare coverage in India: a call to action. Value in Health Regional Issues. 2020 May 1;21:226-9.
16. National Cost database.National Health System Cost Database for India. Access on https://www.healtheconomics.pgisph.in/costing_web/
17. Briggs A, Sculpher M, Claxton K. Decision modelling for health economic evaluation. Oup Oxford; 2006 Aug 18.
18. Drummond MF, Sculpher MJ, Claxton K, Stoddart GL, Torrance GW. Methods for the economic evaluation of health care programmes. Oxford university press; 2015 Sep 25.
19. Glick HA, Doshi JA, Sonnad SS, Polsky D. Economic evaluation in clinical trials. OUP Oxford; 2014 Oct 2.

For peer review only

18

For peer review only

BMJ Open

A Study Protocol for Economic Evaluation of Probiotic Intervention for Prevention of Neonatal Sepsis In 0-2 Month Old Low Birth Weight Infants In India - The ProSPoNS Trial

Journal:	*BMJ Open*
Manuscript ID	bmjopen-2022-068215.R1
Article Type:	Protocol
Date Submitted by the Author:	24-Jan-2023
Complete List of Authors:	sinha, anju; Indian Council of Medical Research, Reproductive and Child Health<br>Bahuguna, Pankaj; Post Graduate Institute of Medical Education and Research School of Public Health, Department of Community Medicine and School of Public Health<br>Gupta, Subodh; MGIMS, Sewagram, Community Medicine; MGIMS, Sewagram, Community Medicine<br>Kuruba, Yamini Priyanka; Indian Council of Medical Research, RBMCH<br>Poluru, Ramesh; The INCLEN Trust International,<br>Mathur, Apoorva; Indian Council of Medical Research, RBMCH<br>Raja, Dilip ; Indian Council of Medical Research, RBMCH<br>Raut, Abhishek; Mahatma Gandhi Institute of Medical Sciences, Department of Community Medicine<br>Mahajan, Kamaleshwar; Mahatma Gandhi Institute of Medical Sciences, Department of Community Medicine<br>Sudhakar, Rishikesh; INCLEN<br>Kulkarni, Bharati; National Institute of Nutrition, ;<br>Pandey, Ravindra Mohan ; All India Institute of Medical Sciences, Statistics<br>Arora, Narendra K. ; The INCLEN Trust International<br>Prinja, Shankar; Post Graduate Institute of Medical Education and Research, Community Medicine and School of Public Health; Post-graduate Institute of Medical Education and Research
**Primary Subject Heading**:	Paediatrics
Secondary Subject Heading:	Health economics
Keywords:	Neonatal intensive & critical care < INTENSIVE & CRITICAL CARE, HEALTH ECONOMICS, Paediatric intensive & critical care < INTENSIVE & CRITICAL CARE

SCHOLARONE™ Manuscripts

Title Page

A Study Protocol for Economic Evaluation of Probiotic Intervention for Prevention of Neonatal Sepsis In 0-2 Month Old Low Birth Weight Infants In India - The ProSPoNS Trial

Anju Pradhan Sinha*[1], Pankaj Bahuguna[2], Subodh S. Gupta[3], Kuruba Yamini Priyanka[1], Ramesh Poluru[4], Apoorva Mathur[1], Dilip Raja Koppula[1], Abhishek V. Raut[3], Kamlesh Mahajan[3], Rishikesh Sudhakar[4], Bharati Kulkarni[1], Ravindra Mohan Pandey[5], Narendra Kumar Arora[4], Shankar Prinja[2]

1. Division of Reproductive, Child Health & Nutrition, Indian Council of Medical Research, New Delhi
2. Department of Community Medicine & School of Public Health, Post Graduate Institute of Medical Education and Research, Chandigarh, India
3. Department of Community Medicine, Mahatma Gandhi Institute of Medical Sciences, Wardha, India
4. The INCLEN Trust International, New Delhi, India
5. Ex. Head of the Department of Biostatistics, All Indian Institute of Medical Science, New Delhi, India

*** Corresponding author details:**

Anju Pradhan Sinha, Indian Council of Medical Research, Reproductive and Child Health, Ansari Nagar, New Delhi-110029, Email ID: apradhandr@gmail.com, Mobile Number: 09811422241

Keywords: Neonatal Sepsis, Low Birth Weight, Economic Evaluation, Probiotics, Disability adjusted life year

Word count (excluding title page, abstract, references, figures and tables): 3960 words

Number of References: 19

Abstract count: 401 words

For peer review only

Abstract

Introduction: There is limited evidence on economic evaluations of interventions for prevention of neonatal sepsis. The ProSPoNS trial is a multicentre, double-blind, placebo-controlled trial to evaluate the role of probiotics in prevention of neonatal sepsis. The present protocol describes the data and methodology for the cost utility of the probiotic intervention alongside the controlled trial.

Methods: A societal perspective will be adopted in the economic evaluation. Direct medical and non-medical costs associated with neonatal sepsis and its treatment would be ascertained in both the intervention and the control arm. Intervention costs will be facilitated through primary data collection and program budgetary records. Treatment cost for neonatal sepsis and associated conditions will be accessed from Indian national costing database estimating healthcare system costs (NHSCD). A cost –utility design will be employed with outcome as incremental cost per disability-adjusted life year (DALY) averted. Considering a time-horizon of 6 months, trial estimates will be extrapolated to model the cost and consequences among high-risk neonatal population in India. A discount rate of 3% will be used. Impact of uncertainties present in analysis will be addressed through both deterministic and probabilistic sensitivity analysis. In addition, budget impact analysis and feasibility analysis will be undertaken to inform affordability and sustainability of the intervention if considered for implementation.

Expected outcomes: This study focuses on evaluating the cost-utility of probiotic intervention for prevention of neonatal sepsis. Findings from the cost-utility analysis will be crucial for induction of the intervention within the ambit of existing governmental programs, if found to be efficacious.

Ethics approval: Has been obtained from EC of the six participating sites (MGIMS Wardha, KEM Pune, JIPMER Pudduchery, AIPH, Bhubaneswar, LHMC New Delhi, SMC Meerut) as well as from the ERC of LSTM, UK.

Strengths:

- This would be the first study evaluating cost effectiveness of using probiotic intervention among low birth weight newborn infants in India.
- This study would inform incremental costs per DALY averted with use of probiotics which will be key for prioritising resource allocation in view of other competing demands for child health, therefore crucial for public policy decisions.

For peer review only

- Data from the RCT would be complimented with the use of a model-based approach to explore scenarios beyond the trial data.

Limitations:

- Generalisability and external validity of findings from economic evaluation concurrently performed with the main study may not represent the same treatment effects and costs as in routine clinical practice.

Introduction

There is progress towards attaining the 3rd Sustainable Development Goal (SDG) of under-five (U5) mortality of 25 per 1000 live births by 2030, in India. Neonatal mortality rate is the most important contributing factor of the U5 mortality [1, 2]. Further decline in neonatal mortality may require newer interventions for prevention or treatment of neonatal infections like meningitis, pneumonia, septicaemia that result in more than a quarter of the 1 million neonatal deaths every year in India [2]. The ProSPoNS trial [3] proposes to evaluate the role of probiotics in prevention of neonatal infections (sepsis) among the vulnerable group of low birth weight infants contributing the highest rates of morbidity and mortality. A larger sample size and precise outcome measures differentiate this trial from the earlier conducted pilot study that showcased a 21% non-significant decline in morbidity due to neonatal sepsis in the intervention arm. Considering the 30% prevalence of low-birth weight (LBW) in India and 30 % mortality due to neonatal sepsis, even a small decline in the incidence of sepsis would translate to a large number of new born lives saved. [3]

If the intervention is proven to be efficacious, it would be important to know whether it is also a cost effective intervention. There are few examples in the literature where economic evaluations have been conducted on other effective interventions to prevent neonatal sepsis.[4,5] It cannot be overemphasized that along with clinical effectiveness of interventions, an estimate of economic effectiveness either in terms of costs or utilities (DALYs and QALYs) is required to make correct choices about their inclusion in public health programs. This is of immense importance in presence of multiple competitive options where prioritization is needed.

Ranjeva et al. [6] have quantified the scope of the public health and economic burden of neonatal sepsis in Sub-Saharan Africa. However, evidence on the health economics aspect of neonatal sepsis prevention effect of probiotics has not been ascertained so far. We propose to evaluate the cost utility of probiotic intervention for prevention of neonatal sepsis within the ProSPoNS trial,

For peer review only

Background

Study Setting & Study population

ProSPoNS trial is a Phase III multicentre randomized, double-blind placebo-controlled trial, being conducted at six study sites across India. It is a global Health trial funded by the UK Research and Innovation (UKRI).

The Study is ongoing at 6 sites across India: Jawaharlal Institute of Postgraduate Medical Education and Research (JIPMER), Puducherry, Mahatma Gandhi Institute of Medical Sciences (MGIMS), Wardha, King Edward Memorial Hospital (KEM Hospital), Pune, Asian Institute of Public Health (AIPH), Bhubaneswar (with recruitments from SCB Medical College and Hospital, Cuttack), Subharti Medical College (SMC) and Lala Lajpat Rai Memorial Medical (LLRM) College District women hospital, Meerut and Lady Hardinge Medical College (LHMC), Kalawati Saran Children's Hospital (KSCH), Delhi. The primary objective is to estimate a 30% reduction in the incidence of neonatal sepsis in the intervention arm with daily supplementation of 1ml (10 billion CFU/ml) of Vivomixx drops over a period of 30 days in 0-2-month-old LBW infants. 6144 Low birth weight (between 1500-2500 g) infants would be recruited to the study from the 6 study sites located in Bhubaneswar, Meerut, New Delhi, Puducherry, Pune, and Sewagram, between ages 3-7 days of life.

Theory of change

More than one-third of the estimated four million neonatal deaths around the world each year are caused by severe infections, and a quarter - around one million deaths - is due to neonatal sepsis/pneumonia alone. Panigrahi et al in their study which was conducted in the year 2017 reported that the Culture confirmed incidence of sepsis as 6.7/1000 births with 51% Gram negatives (Klebsiella predominating) and 26% Gram positives (mostly Staphylococcus aureus). The Sustainable Development Goal for child survival cannot be achieved without substantial reductions in infection-specific neonatal mortality [1]. Due to non-specific signs and symptoms, sepsis is difficult to diagnose. The chances of recovery and survival depend on the correct diagnosis, referral, reaching a facility and adequate treatment by appropriately-trained health workers and high-quality services.

Low Birth Weight is one of the important indirect causes of deaths in neonates accounting for 40-80% of neonatal deaths worldwide [2]. LBW neonates are immune compromised and manifest poor cognitive functions [7]. Since LBW infants are more prone to infections, neonatal mortality and morbidity may be decreased by preventing neonatal infection.

For peer review only

Antibiotic therapy is the mainstay of current therapeutic management of neonatal infections. For serious infections in new borns and young infants, there is a lack of guidelines on rational antibiotic use [8]. In the absence of rapid diagnostics, non-judicious use of antibiotics contributes to the rising antimicrobial resistance. Antibiotic overuse is linked with disturbing the gut ecosystem. The problem of drug resistance outweighs the fast pace of newer generation antibiotic production.

Currently, there are no preventive interventions available for neonatal sepsis other than general measures of hand washing, exclusive breastfeeding, etc. However evaluating immunotherapy with immune globulin, probiotics, myeloid colony-stimulating factors, glutamine supplementation, recombinant human protein C and lactoferrin are being evaluated as adjuvants for the prevention of neonatal sepsis [9]. Probiotics are non-pathogenic microbes which are capable of exerting positive health effects in the host body through various mechanisms [10] [11]. It acts by suppressing the growth of potential pathogens and their epithelial attachment. It also works by secreting anti-microbial substances or by stimulating host expression of protective molecules. Besides, it can produce a barrier against the harmful pathogens at higher levels. It stimulates the process of immunosuppressive agent's production in the host which suppress the inflammatory responses or accelerate the immunologic mechanism that clears the infection. Probiotics stimulate normal microbe-microbe and host-microbe interactions positively, thereby enhance the protection provided by commensal flora through competitive interactions, pathogenic antagonism, and antimicrobial factors. We hypothesize that immunomodulation/ immunopotentiation with probiotics may prove to be an alternative for the prevention of neonatal sepsis. **Figure 1** depicts the conceptual framework to prevent sepsis episodes among 0-2 months neonatal population with use of probiotics.

Research Question:

Is probiotic (vivomax) use as prophylaxis a cost-effective strategy for preventing sepsis among 0-2 month old infant population in India?

Objectives: To assess the cost utility of using probiotics as prophylaxis for sepsis among LBW neonates of age 0 to 2 months.

Study Methodology

A cost utility analysis is proposed to be conducted alongside the multicentre RCT. Randomized controlled trials (RCT) are considered the 'gold standard' as they produce a reliable the best evidence of treatment effect. The economic evaluation alongside RCT provides opportunity to

For peer review only - http://bmjopen.bmj.com/site/about/guidelines.xhtml

collect data both on disease-specific disability and resource use. Start date is 15th November 2022 and the anticipated completion date is December 2023

Model Overview

General Description:

This protocol describes the methodology for economic evaluation within a phase III RCT – the ProSPoNS trial. The details are described in the following sections.

Decision Model

In view of the shortcomings of treatment effect measure coming from a single RCT, we also propose using a model-based approach which will allow synthesizing information from all sources for a health technology enabling us to explore scenarios not explicitly found in the trial data. Moreover, the modelling approach would enable to estimate the long-term benefits of intervention incorporating quality of life measures. Furthermore, it allows accounting for uncertainties present in assumptions and data used for economic evaluation, which has implications for policy question under consideration. The clinical outcomes from trial would be sepsis episodes averted as an effect of probiotics use in the intervention arm. Quality of life (QUALY) is a preferred utility measure for economic analysis, however there is no EQ-5D version for neonates. Therefore, in order to assess the long-term effects, we propose to use 'Disability adjusted life years' (DALY) as the measure of health outcome which accounts for both disability and quality of life. A decision-analytic model following a systematic approach in bringing together evidence on clinical effectiveness, health-related disability of life and costs associated with the probiotic intervention as compared to placebo will be used with the aim to provide information on whether the reduction in neonatal sepsis incidence justifies the incremental costs. **Figure 2** depicts the decision model for the proposed study.

Outcome measures: Primary outcome measure for economic evaluation (within-trial CUA) would be sepsis episodes and related deaths averted in the intervention arm as compared to the placebo arm, which translates to gain in life years and DALYs averted as long-term measure.

Data requirements: Data requirements for economic evaluation is tabulated in Table-1.

Table- 1: Data requirements for economic evaluation:

**Data Type**	**Significance for the Model**	**Source of Data**
Demographic	- Estimate the baseline parameters	Primary data collection; Census of India

For peer review only

	- Estimate target population	
Treatment effects & Incidence of Adverse Events	- Efficacy and safety parameters<br>- Cost estimation<br>- Outcome assessment	RCT Database
Background Mortality data	- Estimate natural mortality in the target population	SRS, Published literature
Utilities	- Estimate DALY as long-term outcome measure	Global Burden of Diseases estimates (2011)
Resource use and price data	- Cost estimation	Primary data collection; NHSC-Prinja et al
Epidemiological data	- Estimate baseline risk of clinical events	Published literature; Primary data collection (Control arm of the RCT)

RCT- Randomised Control Trial, SRS- Sample Registration System, NHSCD- National Health Systems Costing Database

Utilities data would allow incorporating the relative impact of a health technology (i.e. probiotics) on the neonatal health outcomes in terms of disability burden. This will be estimated as DALYs averted. Utility data would be obtained from Global disease burden estimates 2017. Cost data are important drivers of cost-effectiveness, as they quantify the resource consumption associated with use of health technologies.

Decision-analytic model would be populated with cost data from the societal perspective: direct medical and non-medical cost borne by patients; and direct medical cost borne by the health care system. A national health system costing database (NHSCD) to estimate health care system's costs in India has been developed. [12] Data on patient's out-of-pocket (OOP) expenses would be collected during the conduct of the RCT as primary data.

A health economic evaluation using cost- utility analysis (CUA) design will be conducted within the ProSPoNS trial. The results would provide the incremental costs and health gains of the probiotic intervention in absence of any prevention modality being implemented or available. Decision-analytical modelling using the trial results would be extrapolated to the target population and time horizon to inform policy on the potential for incremental costs offsets attributable to prevention of morbidity with the proposed intervention. The CUA will use the present results as cost per case of

For peer review only

neonatal sepsis prevented. CUA will present the results as cost per disability-adjusted life year averted. Intervention costs and effects will be extrapolated to the Indian neonatal population. Modelling will be used for the target population, time horizon, and the decision context to provide much required information for the policymakers. It would guide further decision for up scaling the intervention, if it is found to be efficacious. If the incremental costs of the intervention can be offset by the gains in terms of effectiveness (i.e. DALYs) it may be presented for induction within the ambit of existing governmental program. Modelled CUA results can be presented as health - adjusted life years saved and savings of the health care system by disease prevention. We will calculate the incremental cost-effectiveness ratios as the difference in the costs between the intervention and the comparator divided by the difference in health outcomes.

Effectiveness assessment:

Prevention of severe sepsis in the early neonates will prevent the long-term morbidity like neurodevelopmental disability risk associated with sepsis [13] and reduce mortality due to pneumonia, meningitis, and neonatal sepsis and hospital admissions due to any infectious diseases [14].

We propose to use the short time horizon to assess the sepsis averted as a result of probiotics supplementation for one month and model the change in DALYs in the intervention group as a long-term measure.

Study details:

Total of 6144 (N) new-born babies with LBW would be screened for eligibility and randomly assigned to probiotic (n=3072) or placebo (n=3072) groups and administered 1 ml of the probiotic suspension daily for 30 days. The study would use IWRS for the purpose of stratified randomization by birth weight, sex and study site. Trained field workers will make home visits daily for first 7 days and thereafter thrice in a week till day-30 of the follow up period to ensure and monitor adherence to the intervention and collect morbidity information. After the 30-day intervention period, home visitation will continue at weekly interval till day-60 of life. During home visitation as per schedule trained fieldworkers would screen infants for symptoms/ signs of PSBI/ Clinical illness/ Clinical severe infection. To record the AE/SAE events (if any), continuous monitoring and reporting of ongoing medical conditions, signs symptoms will be conducted by field worker during their scheduled follow-up home visits using software on handheld device during the two-months period of follow up. Presence of any danger signs would be evaluated, and need for referral would be assessed: If a child is found to have any symptoms/ signs from the list, the field worker will accompany the

For peer review only

infant to facility for examination by study physician (referral). The study physician would examine the infant again for symptoms/ signs of PSBI/ Clinical illness/ Clinical severe infection and confirm the diagnosis. Investigations such as blood culture, sepsis screen would be performed for suspected cases of neonatal sepsis by study physician. If required the infant would be hospitalized for treatment. Data on outcomes, lab investigation and treatment during clinical management of the sick new-born will be collected and serious adverse events will be reported as per regulatory guidelines. Blood culture isolates will be shipped by site to microbiology lab at All India Institute of Medical Sciences (AIIMS), New Delhi for quality assurance purpose.

The study will be implemented according to MRC guidelines for management of global health trials and will be governed by a Trial Steering Committee and a Data Management Committee.

Primary outcomes:

1. Sepsis: defined as one or more clinical symptoms suggestive of sepsis with a microbial isolate on blood culture; or a neonate with sterile blood culture with at least 2 sepsis screen markers being abnormal (Absolute neutrophil count<1500/mm3; TLC<5000/mm3; Immature to total neutrophil ratio > 0.2; Immature to total neutrophil ratio > 0.2;CRP >1.2 mg/dl, ESR>15 mm).

2. Possible Serious Bacterial Infection (PSBI): One or more clinical signs like fever (temperature ≥ 38 °C), low body temperature (< 35.5 °C), not feeding well, convulsions, fast breathing (60 breaths per minute or more), severe chest in-drawing, movement only when stimulated or no movement at all in infants less than 7 days old confirmed by study physician.

Secondary outcomes:

1. Clinical severe infection: One or more clinical signs like severe chest in-drawing, not feeding well, fever (temperature ≥ 38 °C), the movement only when stimulated as confirmed by the study physician, low body temperature (< 35.5 °C).

2. Critical Illness: One or more of clinical signs like unable to feed at all, unable to cry, no movement on stimulation, bulging fontanelle, cyanosis as confirmed by the study physician and convulsions. The flow chart below (Figure 1) depicts the study design and the study processes that will be followed during the implementation of the proposed research work (Diagram, to be finalised after finalisation of methods). Primary and secondary outcomes would be compared between the two study arms to assess clinical effectiveness of probiotic intervention.

For peer review only

Costing

In the current study, cost of Vivomix (intervention), cost of routine home-based postnatal care (HBPNC), cost of illness management, drug costs, vitamin/mineral supplement costs, laboratory investigation charges, and doctor consultation, bed charges are categorized as direct medical costs and the informal payments and travel are categorized as the direct non-medical costs. Loss of wages of parents and other caregivers due to absenteeism from work and is categorized as indirect costs. Neonatal sepsis episodes are treated in in-patient setting; therefore respondents (parents and caregivers) will be interviewed to collect information on days they were absent from work due to hospital stay for treatment. After hospital discharge, follow up with parents and caregivers to assess any effect on their productivity at work is beyond the scope of data collection in the present study. All costs related data would be collected from all six study sites in a representative manner.

Sample size (Costing): We calculated the sample size to estimate the mean out-of-pocket (OOP) expenditures among the neonates with illness (here PSBI). A power of 80% and 5% level of significance was assumed for sample size calculation. The baseline estimate for OOP among neonates with PSBI was accessed from an Indian study. [1] Assuming a mean OOP of □2003 (Std deviation: □3517) for neonates with PSBI and an error margin of 7.5%, the minimum sample size computed to estimate the OOP was 1998. In view of high variability in the OOP data, the error margin of 7.5% was considered appropriate for sample size calculation. The data on OOP will be captured for a sub-sample i.e. 1998 neonates, out of total sample recruited in the study. At each of the six subjects recruitment sites data would be collected on 333 subjects for this sub-study. The OOP data will be collected irrespective of status of neonates belonging to two study arms. The formula used for sample size calculation is given below:

$$n = \frac{Z^2_{1-a/2}\, s^2}{d^2}$$

Where; n= minimum estimated sample size

Z^2_{1-a}= table value from standard normal distribution

s^2= standard deviation of outcome variable in study population

d^2 = error margin (or absolute precision)

For peer review only

Data collection & Estimation of costs: Data collection for the current study would be completed in one year duration from September 2022 to August 2023 (to cover any variation with seasonality). Data on out-of-pocket expenditures (OOP) will be collected on a sub-sample of 333 subjects drawn from the total trial population in a representative manner.

Socio- demographic data on education, occupation of parents of enrolled children is being collected on study forms. Parents would be asked about any illness during the study period and any expenditures on account of care seeking, visits to facilities, hospitalization and treatment. Direct medical and non-medical costs due to neonatal sepsis would be ascertained in all 333 subjects at the six study sites. Data on OOP expenditure in public or private health facilities would be elicited.

Intervention cost would be facilitated through primary data collection and the program budgetary records. The cost of treatment for neonatal sepsis and related diseases at secondary and tertiary level will be ascertained from the NHSCD[15,16] and published literature in Indian context.[12] These sources provides estimates on cost per in-patient (IP) admission, cost of intensive care unit (ICU) admission, cost per out-patient (OP) consultation accounting for resources consumed for service provision such as medical (drugs, diagnostics, procedural, hospital charges) and non-medical (transportation, boarding & lodging). In addition, cost related to IEC material, monitoring & supervision and other administrative costs will also be estimated. Assumptions made in the study protocol are provided in Table 2.

Table 2: Key methods and assumptions for economic evaluation:

Component	Consideration/Approach	Justification
Intervention	- Vivomixx (8 strain probiotics) + Sepsis management<br>- Dose: 1ml/day (10 billion CFU/ml) for 30 days<br>- Intervention delivery: Under the supervision of study staff (field worker)	Effectiveness of Vivomixx for preventing neonatal sepsis is being assessed under trial
Comparator	- Placebo	- Effectiveness of Vivomixx is being assessed under trial against Placebo<br>- Use of antibiotic is routine practice for sepsis management.
Target Population	- High-risk LBW neonates (Both pre-term and small for-date babies)	Most vulnerable high-risk population with high morbidity & mortality
Objective	- To assess the incremental cost per	Besides effectiveness, evidence on

For peer review only

	DALY averted with use of probiotics for prevention of neonatal sepsis compared the placebo	cost-effectiveness is mandatory for adoption of treatment/intervention under public policy
Target Audience/Policy Use	- National Health Mission (NHM)<br>- Any other	This intervention is to be implemented under RMNCH program
Method	- Both model and trial based	- Short –term outcome estimates will be used for trial<br>-Use of model-based CEA will enable us to assess long-term outcomes (and sub-group analysis)
Perspective	- Societal perspective	OOP burden associated with sepsis management among LBW neonates
Analytic Horizon	- 6 months	To model long-term outcomes (DALYs)
Costs	- Health system costs (Vivomixx cost; Cost of routine HBPNC care; cost of illness management etc.)<br>- OOP expenditures (Doctor's consultation; bed charges; Drug costs; Vitamin/Mineral supplement costs; Investigations; Procedures; Informal payments; Travel cost etc.	Both health system and patients' costs as per societal perspective considered for CEA
Outcomes	- Sepsis episodes and deaths averted<br>- DALYs<br>- Hospitalization averted<br>- Life years gained	In line with study design (cost-effectiveness/cost-utility analysis)
Discount rate	- 3%	As per Indian reference case

Total cost will be comprised of cost of intervention, cost of management of sepsis and associated complications and patient costs as OOP. Similarly, total cost in the control arm will be estimated in the absence of intervention. Annual discount rate of 3% will be used wherever applicable. In the present study both model based approach as well as trial data would be used. Data management and analysis will be done using MS-Excel.

For peer review only

Other Analyses: Uncertainty analyses, Budget impact analysis, Stakeholder analysis would be included.

Uncertainty Analysis: Both deterministic and probabilistic sensitivity analysis (PSA) will be undertaken to address the effect of uncertainties present in analysis. For PSA, all the parameter values will be varied within a plausible range. Based on nature of parameter, an appropriate probabilistic distribution will be assigned such as beta distribution for risk and utility parameters, gamma distribution for cost parameters, uniform distribution for demographic parameters etc. The results under PSA will be simulated 999 times to report a 95% confidence interval for ICERs.

Budget Impact Analysis: A Budget impact analysis is proposed to be conducted to estimate the financial consequences of increasing uptake of probiotic supplementation for LBW infants in the neonatal care under NHM, over the near future (3 to 5 years). Data regarding the target population of LBW infants will be available by extrapolating the data from the RCT, as will be other epidemiological inputs to populate the budget-impact model. At present there are no alternative prevention modalities for neonatal sepsis present (other than hand hygiene and exclusive breastfeeding). Therefore, assumption would be made regarding the uptake of probiotic supplementation (based on the results of the study).

Stakeholder analysis: A range of potential stakeholders will be consulted, involving experts such as neonatologists/paediatricians, academics, policy makers, program managers, industry, patient group representatives etc. Multiple consultations with stakeholders will be done during the conduct of study as well as dissemination of study results.

Patient and Public Involvement: Patients or caregivers or members of public were not involved in the development of this protocol

Discussion

The ProSPoNS trial is evaluating the role of probiotics intervention in prevention of neonatal infections, a major cause for neonatal mortality. To date no prevention modalities other than hand hygiene and exclusive breastfeeding have been successfully incorporated in programs for prevention of neonatal infections. Panigrahi el al (2017) established benefit of Lactobacillus planitarum with fructooligosaccharides intervention in prevention of sepsis and related deaths in new-borns; however, the study did not assess the cost effectiveness of the intervention. There is no study in the literature

For peer review only

evaluating the cost-effectiveness of this novel disease prevention technology. We have proposed such evaluation alongside the ProSPoNS trial.

A RCT design, considered a gold standard for such evaluations is being used in the trial. However, there are disadvantages of using RCT data quoted in the literature. Data from a single RCT may not be adequate for estimation of cost utility as RCTs mainly focus on a single intervention and have small follow up period, thus, lack to provide evidence on effectiveness of multiple alternatives and situations beyond the trial conditions. The treatment effect measured in a single study may be relevant for a particular setting but has limited generalizability of results in populations other than the one studied. [17, 18, 19]. We propose using a modelling approach to overcome the above mentioned shortcomings of RCT data.

There are a few examples of estimation of cost-effectiveness of disease preventive technologies in the neonatal population. A study conducted in Sub-Saharan Africa estimated that neonatal sepsis resulted in 5.29-8.73 DALYs lost annually accounting for an economic burden from $10 billion to $469 billion (per annum). It was recommended that a strategic plan for the treatment and prevention of neonatal sepsis may reduce this burden.[6] A cost-effective analysis conducted in Ghana reported that alcohol-based hand rub (ABH) effectively reduced the patient cost of neonatal bloodstream infection (BSI), Hospital cost associated with BSI, length of hospital stay, and deaths due to BSI by 41.7%, 48.5%, 50% and 73% respectively. It was also estimated that ABH reduced the incidence of hospital-acquired neonatal infections by 16%.[4]. Current study would estimate the cost-effectiveness of a novel neonatal sepsis prevention modality.

In considering cost-effectiveness, measures on treatment effect over longer time horizon than RCTs are often required. Therefore we have proposed a modelling approach in addition to the use of primary data coming from the ongoing RCT. This approach would use and provide a wider range of assumptions/parameters from multiple data sources and evaluate the long term utility of the probiotic intervention in disease prevention.

Expected outcomes of study and policy implications:

The health economics evaluation would generate data on cost involved with the new intervention as compared to the control arm with uncertainty range, Incremental costs, and health outcomes for both study arms with uncertainty range, ICER, sensitivity and threshold analyses. It would be helpful for the policy maker to understand the cost utility of the probiotic intervention in presence of other competing priorities.

Strengths:

For peer review only

- This would be the first study evaluating cost effectiveness of using probiotic intervention among low birth weight newborn infants in India.
- This study would inform incremental costs per DALY averted with use of probiotics which will be key for prioritising resource allocation in view of other competing demands for child health, therefore crucial for public policy decisions.
- Data from the RCT would be complimented with the use of a model-based approach to explore scenarios beyond the trial data.

Challenges:

- Generalisability and external validity of findings from economic evaluation concurrently performed with the main study may not represent the same treatment effects and costs as in routine clinical practice.

Registration: The protocol has been approved by the regulatory authority (Central Drugs Standards Control Organisation; CDSCO) in India (CT-NOC No. CT/NOC/17/2019 dated 01 March 2019). The ProSPoNS trial is registered at the Clinical Trial Registry of India (CTRI). CTRI/2019/05/019197. Registered on 16 May 2019

Ethics and Dissemination: Approval has been obtained from EC of each of the six participating sites (MGIMS Wardha, KEM Pune, JIPMER Pudduchery, AIPH, Bhubaneswar, LHMC New Delhi, SMC Meerut) as well as from the ERC of LSTM, UK. A peer reviewed article will be published after completion of the study. Findings will be disseminated to in the community of the study sites, with academic bodies and policy makers.

This manuscript is in compliance with the SPIRIT checklist (Research Checklist file)

Author affiliations: Anju Pradhan Sinha[1], Pankaj Bahuguna[2], Subodh S. Gupta[3], Kuruba Yamini Priyanka[1], Ramesh Poluru[4], Apoorva Mathur[1], Dilip Raja Koppula[1], Abhishek V. Raut[3], Kamlesh

For peer review only

Mahajan[3], Rishikesh Sudhakar[4], Bharati Kulkarni[1], Ravindra Mohan Pandey[5], Narendra Kumar Arora[4], Shankar Prinja[2] & the ProSPoNS Trial group of investigators.

6. Indian Council of Medical Research, New Delhi
7. Post Graduate Institute of Medical Education and Research, Chandigarh
8. Mahatma Gandhi Institute of Medical Sciences, Wardha
9. INCLEN Trust International, New Delhi
10. All Indian Institute of Medical Science, New Delhi

Author Contributions:

Conceptualization: Anju Pradhan Sinha, Subodh S. Gupta, Narendra Kumar Arora, Abhishek V. Raut, Ramesh Poluru.

Study design, tools and analysis plan: Pankaj Bahuguna, and Shankar Prinja, Kuruba Yamini Priyanka, Apoorva Mathur, Dilip Raja Koppula.

Writing- review & editing: Bharti Kulkarni, Subodh S. Gupta, Anju Pradhan Sinha, Kamlesh Mahajan, Rishikesh Sudhakar, Ravindra Mohan Pandey, Narendra Kumar Arora.

Writing-original draft: Anju Pradhan Sinha, Pankaj Bahuguna, Kuruba Yamini Priyanka**.**

List of Authors: ProSPoNS Trial group of investigators Ashish Bavdekar, Sunil Sazawal, Sushma Nangia, Sailaja Nandan Parida, B. Adhsivam.

Funding: The study is funded by the Department of Health and Social Care (DHSC), the Foreign, Commonwealth & Development Office (FCDO), the Medical Research Council (MRC) and Wellcome. Award/grant number is MR/S004912/1. The funding body has no role in the designing of the study and collection, analysis, and interpretation of data and in writing the manuscript.

Conflicts of Interests: The Authors declare that they have no conflicts of Interests.

Data Statement: As this is a study protocol at this point, we can’t provide data statement.

Acknowledgements: We would like to thank the Indian Council of Medical Research (ICMR) for the permission to conduct the study and the UK Research and Innovation, Medical Research

Council, UK, for funding the trial under the Global Health Trials [JGHT] scheme 8, jointly funded by the DFID/NIHR/MRC/Wellcome Trust. We thank Dr. Soumya Swaminathan, Ex-Director General of the Indian Council of Medical Research (ICMR), New Delhi and late Prof. M.K. Bhan, for their motivation, encouragement and support with this study. The authors also thank M/s Next Gen Pharma India Pvt. Ltd., India, for being the trial partners (supply of the Active Pharmaceutical Ingredient (API)) and M/s Cadila Healthcare Ltd., India, for agreeing to manufacture and supply the finished formulation: probiotic drops and corresponding placebo used in the study. The authors especially thank the Liverpool School of Tropical Medicine Research Ethics Committee (LSTM REC), IEC of each participating site (participant recruitment site and laboratory site), the Data Monitoring Committee, and the Trial Steering Committee (TSC) for their review and inputs.

For peer review only

References:

1. India Newborn Action Plan (INAP): National Health Mission [Internet]. [Cited 2021 Mar 10]. Available from: https://nhm.gov.in/index4.php?lang=1&level=0&linkid=153&lid=174 https://www.newbornwhocc.org/INAP_Final.pdf.
2. Sachs JD. From millennium development goals to sustainable development goals. Lancet. 2012; 379(9832):2206–11. https://doi.org/10.1016/S0140-673 6(12)60685-0.
3. Sinha AP, Gupta SS, Poluru R, Raut AV, Arora NK, Pandey RM, Sahu AR, Bethou A, Sazawal S, Parida S, Bavdekar A. Evaluating the efficacy of a multistrain probiotic supplementation for prevention of neonatal sepsis in 0–2-month-old low birth weight infants in India—the "ProSPoNS" Study protocol for a phase III, multicentre, randomized, double-blind, placebo-controlled trial. Trials. 2021 Dec; 22(1):1-2.
4. Fenny AP, Otieku E, Labi KA-K, Asante FA, Enemark U (2022) Cost-effectiveness analysis of alcohol handrub for the prevention of neonatal bloodstream infections: Evidence from HAI-Ghana study. PLoS ONE 17(3): e0264905.
5. Nice Clinical Guideline 2012 on Antibiotic for early-onset neonatal infection.
6. Ranjeva SL, Warf BC, Schiff SJ. Economic burden of neonatal sepsis in sub- Saharan Africa. BMJ Glob Health2018; **3:e000347**. doi:10.1136/ bmjgh-2017-000347
7. Allen L and Gillespie S. What works? A review of the efficacy and effectiveness of nutrition interventions. ACC/SCN Nutrition Policy Paper No 19, ADB Nutrition and Development series No 5, 2001: 5-22.
8. Lin HC, Su BH, Chen AC, et al. Oral probiotics reduce the incidence and severity of necrotizing enterocolitis in very low birth weight infants. Paediatrics 2005; 115(1): 1-4
9. Wolkowiez MC, Benjamin DK, Capparelli E. Immunotherapy in neonatal sepsis: Advances in treatment and prophylaxis. Curr Opin Pediatr 2009; 21(2):177-181.
10. MarteanP, Seksik P, Jian R Probiotics and intestinal health effects: a clinical perspective. Br J. Nutr;2002,Sep; 88 Suppl 1:S51-7
11. R. Balfour Sartor, Probiotic therapy of intestinal inflammation and infections. Current opinion in Gastroenterology 2005, 21:44-50
12. Prinja S, Brar S, Singh MP, et al. Process evaluation of health system costing - Experience from CHSI study in India. PLoS One. 2020; 15(5):e0232873. Published 2020 May 13. doi:10.1371/journal.pone.0232873

13. Cai S, Thompson DK, Anderson PJ, Yang JY. Short- and Long-Term Neurodevelopmental Outcomes of Very Preterm Infants with Neonatal Sepsis: A Systematic Review and Meta-Analysis. Children 2019, 6, 131; doi:10.3390/children6120131
14. Wiens MO, Kissoon N, Holsti L. Challenges in pediatric post-sepsis care in resource limited settings: a narrative review. Transl Pediatr 2021; 10(10):2666-2677.
15. Prinja S, Chauhan AS, Rajsekhar K, Downey L, Bahuguna P, Sachin O, Guinness L. Addressing the cost data gap for universal healthcare coverage in India: a call to action. Value in Health Regional Issues. 2020 May 1;21:226-9.
16. National Cost database.National Health System Cost Database for India. Access on https://www.healtheconomics.pgisph.in/costing_web/
17. Briggs A, Sculpher M, Claxton K. Decision modelling for health economic evaluation. Oup Oxford; 2006 Aug 18.
18. Drummond MF, Sculpher MJ, Claxton K, Stoddart GL, Torrance GW. Methods for the economic evaluation of health care programmes. Oxford university press; 2015 Sep 25.
19. Glick HA, Doshi JA, Sonnad SS, Polsky D. Economic evaluation in clinical trials. OUP Oxford; 2014 Oct 2.

Figure legends:

Fig.1 Conceptual framework to prevent neonatal sepsis using probiotics.

Fig.2. Decision tree for cost effectiveness of using probiotics as prophylaxis for sepsis among neonates of age 0 to 2 months.

For peer review only

Annexures

Annexure 1: List of abbreviations

1. EC: Ethics Committee
2. ERC: It is inadvertently mentioned as ERC in the manuscript; however, it is REC which means Research Ethics Committee (REC) of the Liverpool School of Tropical Health, (LSTM) UK
3. MRC: The Medical Research Council
4. UKRI: United Kingdom Research and Innovation
5. NHSCD: The National Health System Cost Database
6. IWRS: Interactive Web Response Systems
7. PSBI: Possible serious bacterial infection.
8. AE/SAE: Adverse events/ Serious adverse events
9. DALY: Disability adjusted life year
10. LMIC: Low- and Middle-income countries
11. SDG: Sustainable Development Goal
12. LBW: Low birth weight.
13. QALY: The quality-adjusted life-year
14. RCT: Randomized controlled trials
15. CUA: Cost utility analysis
16. SRS: Sample Registration System
17. NHSCD- National Health Systems Costing Database
18. OOP: out-of-pocket
19. TLC: Total Leukocyte Count
20. CRP: C-reactive protein.
21. ESR: Erythrocyte sedimentation rate
22. HBPNC: home based post-natal care
23. NHM: National Health Mission
24. RMNCH: Reproductive, Maternal, New-born, Child, and Adolescent Health
25. CEA: Cost-effectiveness analysis
26. PSA: Probabilistic sensitivity analysis.
27. NHM: National Health Mission
28. ABH: Alcohol-based hand rub
29. BSI: Blood stream infection
30. IECR: Incremental cost-effectiveness ratio
31. CDSCO: Central Drugs Standards Control Organisation
32. IEC: Institutional Ethics Committee
33. EQ-5D: EuroQol- 5 Dimension

For peer review only

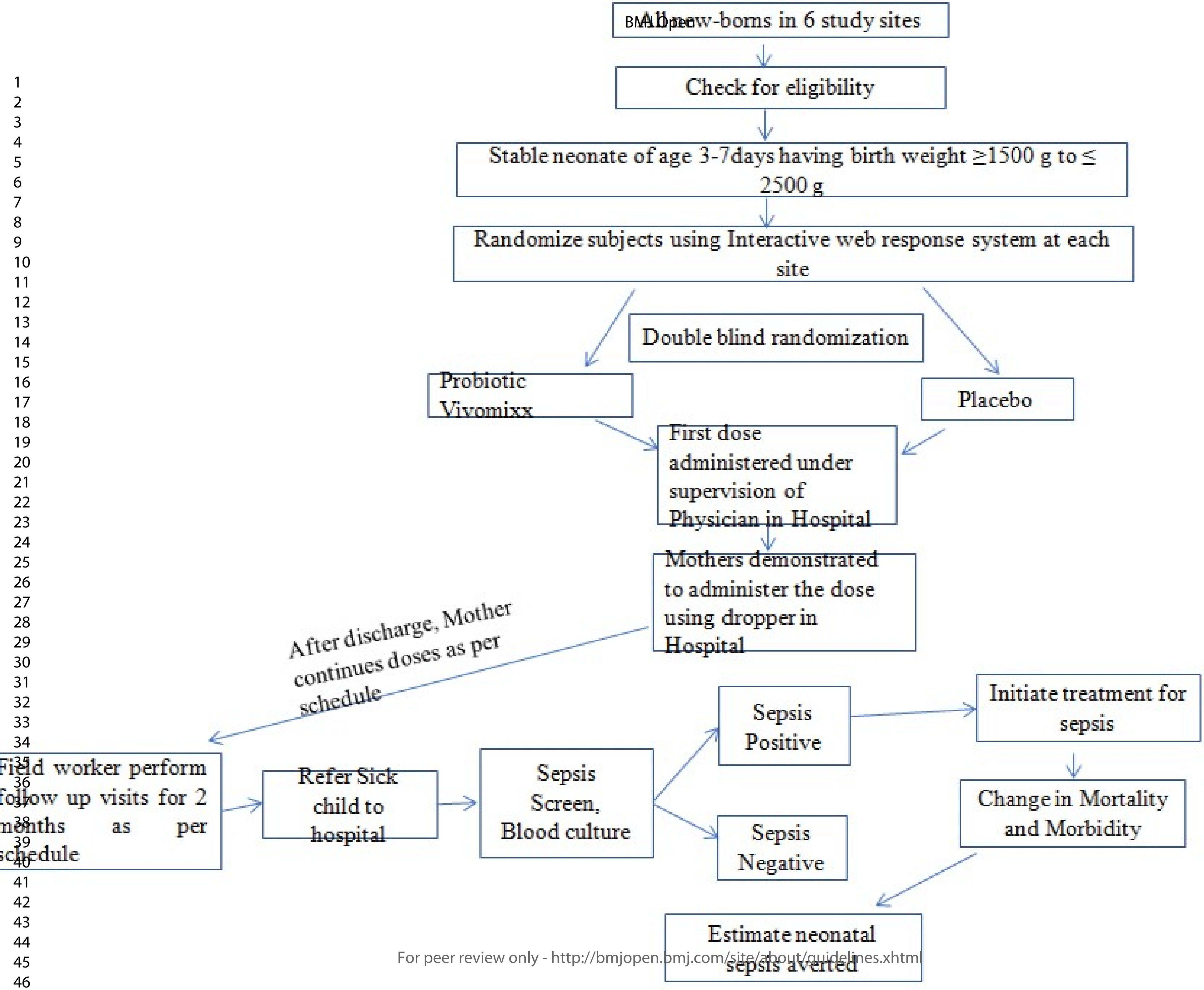
All new-borns in 6 study sites
Check for eligibility
Stable neonate of age 3-7days having birth weight ≥1500 g to ≤ 2500 g
Randomize subjects using Interactive web response system at each site
Double blind randomization
Probiotic Vivomixx
Placebo
First dose administered under supervision of Physician in Hospital
Mothers demonstrated to administer the dose using dropper in Hospital
After discharge, Mother continues doses as per schedule
Field worker perform follow up visits for 2 months as per schedule
Refer Sick child to hospital
Sepsis Screen, Blood culture
Sepsis Positive
Sepsis Negative
Initiate treatment for sepsis
Change in Mortality and Morbidity
Estimate neonatal sepsis averted

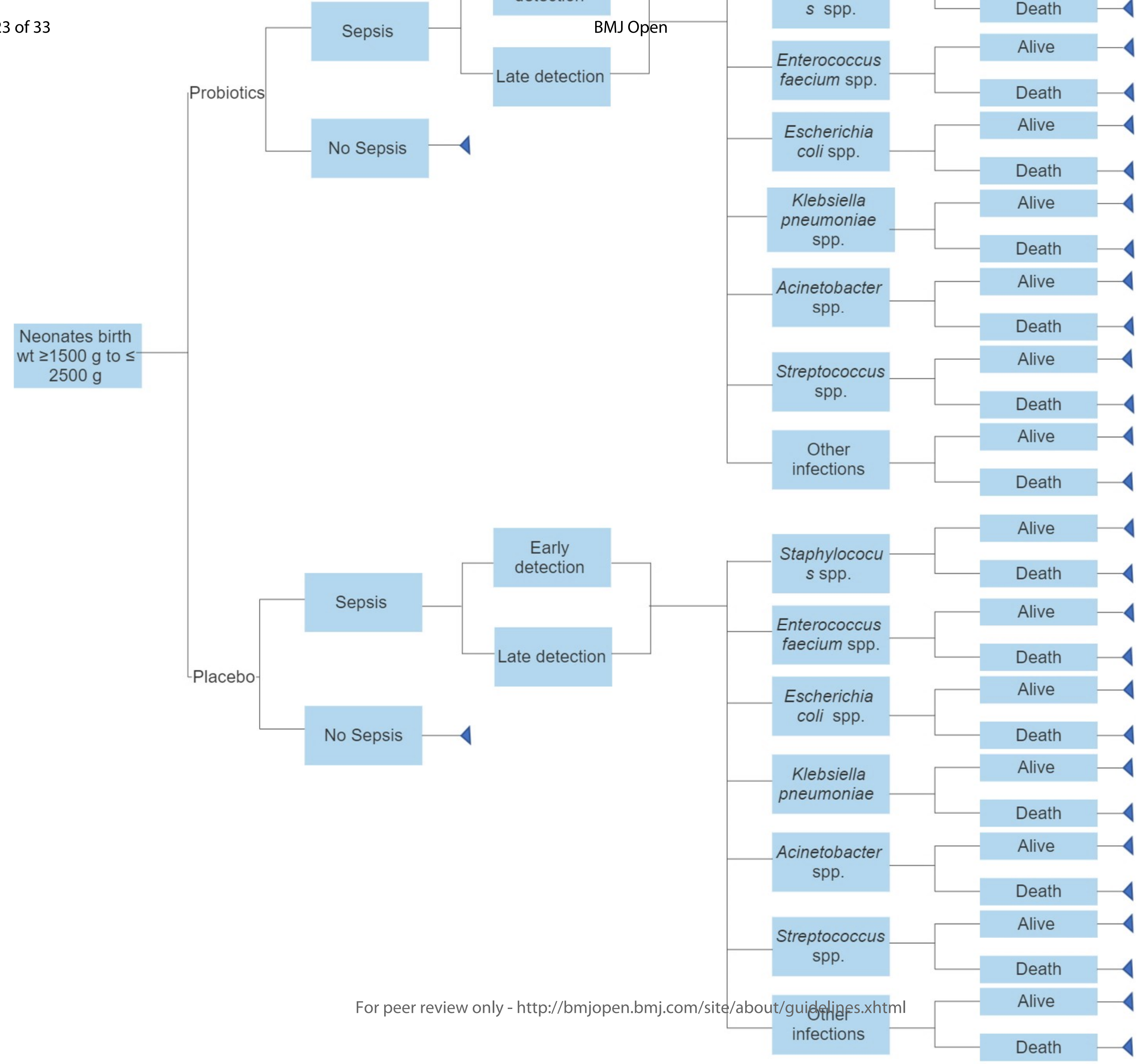
Neonates birth wt ≥1500 g to ≤ 2500 g
Probiotics
Sepsis
Late detection
No Sepsis
s spp.
Enterococcus faecium spp.
Escherichia coli spp.
Klebsiella pneumoniae spp.
Acinetobacter spp.
Streptococcus spp.
Other infections
Alive
Death
Placebo
Sepsis
Early detection
Late detection
No Sepsis
Staphylococcus spp.
Enterococcus faecium spp.
Escherichia coli spp.
Klebsiella pneumoniae
Acinetobacter spp.
Streptococcus spp.
Other infections

SPIRIT
STANDARD PROTOCOL ITEMS: RECOMMENDATIONS FOR INTERVENTIONAL TRIALS

SPIRIT 2013 Checklist: Recommended items to address in a clinical trial protocol and related documents*

**Section/item**	**ItemNo**	**Description**	**Page number and line number**
**Administrative information**			
Title	1	Descriptive title identifying the study design, population, interventions, and, if applicable, trial acronym	Page No. 1<br>Line No. 3-5
Trial registration	2a	Trial identifier and registry name. If not yet registered, name of intended registry	Page No. 15<br>Line No. 6-9
	2b	All items from the World Health Organization Trial Registration Data Set	Trial was registered in CTRI which is govern by WHO
Protocol version	3	Date and version identifier	Page No. 15<br>Line No. 6-9
Funding	4	Sources and types of financial, material, and other support	Page No. 16<br>Line No. 7-11
Roles and responsibilities	5a	Names, affiliations, and roles of protocol contributors	Page No. 15 Line No. 16-29<br>Page No. 16 Line No. 1-3
	5b	Name and contact information for the trial sponsor	Page No. 16<br>Line No. 7-11
	5c	Role of study sponsor and funders, if any, in study design; collection, management, analysis, and interpretation of data; writing of the report; and the decision to submit the report for publication, including whether they will have ultimate authority over any of these activities	No

For peer review only

	5d	Composition, roles, and responsibilities of the coordinating centre, steering committee, endpoint adjudication committee, data management team, and other individuals or groups overseeing the trial, if applicable (see Item 21a for data monitoring committee)	Described in the below given study protocol at Page No.3 Sinha AP, Gupta SS, Poluru R, Raut AV, Arora NK, Pandey RM, Sahu AR, Bethou A, Sazawal S, Parida S, Bavdekar A. Evaluating the efficacy of a multistrain probiotic supplementation for prevention of neonatal sepsis in 0–2-month-old low birth weight infants in India—the "ProSPoNS" Study protocol for a phase III, multicentric, randomized, double-blind, placebo-controlled trial. Trials. 2021 Dec;22(1):1-2.
**Introduction**			
Background and rationale	6a	Description of research question and justification for undertaking the trial, including summary of relevant studies (published and unpublished) examining benefits and harms for each intervention	Page No. 5<br>Line No. 4-23
	6b	Explanation for choice of comparators	Not applicable
Objectives	7	Specific objectives or hypotheses	Page No. 5<br>Line No. 24-25
Trial design	8	Description of trial design including type of trial (eg, parallel group, crossover, factorial, single group), allocation ratio, and framework (eg, superiority, equivalence, noninferiority, exploratory)	Page No. 8<br>Line No. 15-18
**Methods: Participants, interventions, and outcomes**			
Study setting	9	Description of study settings (eg, community clinic, academic hospital) and list of countries where data will be collected. Reference to where list of study sites can be obtained	Page No. 4<br>Line No. 2-12

For peer review only - http://bmjopen.bmj.com/site/about/guidelines.xhtml

Eligibility criteria	10	Inclusion and exclusion criteria for participants. If applicable, eligibility criteria for study centres and individuals who will perform the interventions (eg, surgeons, psychotherapists)	Described in the below given study protocol at Page No.5-6<br>Sinha AP, Gupta SS, Poluru R, Raut AV, Arora NK, Pandey RM, Sahu AR, Bethou A, Sazawal S, Parida S, Bavdekar A. Evaluating the efficacy of a multistrain probiotic supplementation for prevention of neonatal sepsis in 0–2-month-old low birth weight infants in India—the "ProSPoNS" Study protocol for a phase III, multicentric, randomized, double-blind, placebo-controlled trial. Trials. 2021 Dec;22(1):1-2.
Interventions	11a	Interventions for each group with sufficient detail to allow replication, including how and when they will be administered	Page No. 8<br>Line No. 15-17
	11b	Criteria for discontinuing or modifying allocated interventions for a given trial participant (eg, drug dose change in response to harms, participant request, or improving/worsening disease)	Not applicable
	11c	Strategies to improve adherence to intervention protocols, and any procedures for monitoring adherence (eg, drug tablet return, laboratory tests)	Page No. 8<br>Line No. 18-21
	11d	Relevant concomitant care and interventions that are permitted or prohibited during the trial	Page No. 8<br>Line No. 27-31
Outcomes	12	Primary, secondary, and other outcomes, including the specific measurement variable (eg, systolic blood pressure), analysis metric (eg, change from baseline, final value, time to event), method of aggregation (eg, median, proportion), and time point for each outcome. Explanation of the clinical relevance of chosen efficacy and harm outcomes is strongly recommended	Page No. 9<br>Line No. 2-23<br>Page No. 11-12<br>Table 2

Participant timeline	13	Time schedule of enrolment, interventions (including any run-ins and washouts), assessments, and visits for participants. A schematic diagram is highly recommended (see Figure)	Described in the below given study protocol at Page No.6 table. 1<br>Sinha AP, Gupta SS, Poluru R, Raut AV, Arora NK, Pandey RM, Sahu AR, Bethou A, Sazawal S, Parida S, Bavdekar A. Evaluating the efficacy of a multistrain probiotic supplementation for prevention of neonatal sepsis in 0–2-month-old low birth weight infants in India—the "ProSPoNS" Study protocol for a phase III, multicentric, randomized, double-blind, placebo-controlled trial. Trials. 2021 Dec;22(1):1-2.
Sample size	14	Estimated number of participants needed to achieve study objectives and how it was determined, including clinical and statistical assumptions supporting any sample size calculations	Page No. 10<br>Line No. 4-23
Recruitment	15	Strategies for achieving adequate participant enrolment to reach target sample size	Described in the below given study protocol at Page No.4<br>Sinha AP, Gupta SS, Poluru R, Raut AV, Arora NK, Pandey RM, Sahu AR, Bethou A, Sazawal S, Parida S, Bavdekar A. Evaluating the efficacy of a multistrain probiotic supplementation for prevention of neonatal sepsis in 0–2-month-old low birth weight infants in India—the "ProSPoNS" Study protocol for a phase III, multicentric, randomized, double-blind, placebo-controlled trial. Trials. 2021 Dec;22(1):1-2.
**Methods: Assignment of interventions (for controlled trials)**			
Allocation:			

Sequence generation	16a	Method of generating the allocation sequence (eg, computer-generated random numbers), and list of any factors for stratification. To reduce predictability of a random sequence, details of any planned restriction (eg, blocking) should be provided in a separate document that is unavailable to those who enrol participants or assign interventions	Page No. 8<br>Line No. 17-18
Allocation concealment mechanism	16b	Mechanism of implementing the allocation sequence (eg, central telephone; sequentially numbered, opaque, sealed envelopes), describing any steps to conceal the sequence until interventions are assigned	Described in the below given study protocol at Page No.7<br>Sinha AP, Gupta SS, Poluru R, Raut AV, Arora NK, Pandey RM, Sahu AR, Bethou A, Sazawal S, Parida S, Bavdekar A. Evaluating the efficacy of a multistrain probiotic supplementation for prevention of neonatal sepsis in 0–2-month-old low birth weight infants in India—the “ProSPoNS” Study protocol for a phase III, multicentric, randomized, double-blind, placebo-controlled trial. Trials. 2021 Dec;22(1):1-2.
Implementation	16c	Who will generate the allocation sequence, who will enrol participants, and who will assign participants to interventions	Described in the below given study protocol at Page No.4<br>Sinha AP, Gupta SS, Poluru R, Raut AV, Arora NK, Pandey RM, Sahu AR, Bethou A, Sazawal S, Parida S, Bavdekar A. Evaluating the efficacy of a multistrain probiotic supplementation for prevention of neonatal sepsis in 0–2-month-old low birth weight infants in India—the “ProSPoNS” Study protocol for a phase III, multicentric, randomized, double-blind, placebo-controlled trial. Trials. 2021 Dec;22(1):1-2.

For peer review only - http://bmjopen.bmj.com/site/about/guidelines.xhtml

Blinding (masking)	17a	Who will be blinded after assignment to interventions (eg, trial participants, care providers, outcome assessors, data analysts), and how	Described in the below given study protocol at Page No.7 Sinha AP, Gupta SS, Poluru R, Raut AV, Arora NK, Pandey RM, Sahu AR, Bethou A, Sazawal S, Parida S, Bavdekar A. Evaluating the efficacy of a multistrain probiotic supplementation for prevention of neonatal sepsis in 0–2-month-old low birth weight infants in India—the "ProSPoNS" Study protocol for a phase III, multicentric, randomized, double-blind, placebo-controlled trial. Trials. 2021 Dec;22(1):1-2.
	17b	If blinded, circumstances under which unblinding is permissible, and procedure for revealing a participant's allocated intervention during the trial	Not applicable
**Methods: Data collection, management, and analysis**			
Data collection methods	18a	Plans for assessment and collection of outcome, baseline, and other trial data, including any related processes to promote data quality (eg, duplicate measurements, training of assessors) and a description of study instruments (eg, questionnaires, laboratory tests) along with their reliability and validity, if known. Reference to where data collection forms can be found, if not in the protocol	Page No. 10 Line No. 25-35<br><br>Page No. 11 Line No. 1-6

	18b	Plans to promote participant retention and complete follow-up, including list of any outcome data to be collected for participants who discontinue or deviate from intervention protocols	Described in the below given study protocol at Page No.4 Sinha AP, Gupta SS, Poluru R, Raut AV, Arora NK, Pandey RM, Sahu AR, Bethou A, Sazawal S, Parida S, Bavdekar A. Evaluating the efficacy of a multistrain probiotic supplementation for prevention of neonatal sepsis in 0–2-month-old low birth weight infants in India—the “ProSPoNS” Study protocol for a phase III, multicentric, randomized, double-blind, placebo-controlled trial. Trials. 2021 Dec;22(1):1-2.
Data management	19	Plans for data entry, coding, security, and storage, including any related processes to promote data quality (eg, double data entry; range checks for data values). Reference to where details of data management procedures can be found, if not in the protocol	Described in the below given study protocol at Page No.8 Sinha AP, Gupta SS, Poluru R, Raut AV, Arora NK, Pandey RM, Sahu AR, Bethou A, Sazawal S, Parida S, Bavdekar A. Evaluating the efficacy of a multistrain probiotic supplementation for prevention of neonatal sepsis in 0–2-month-old low birth weight infants in India—the “ProSPoNS” Study protocol for a phase III, multicentric, randomized, double-blind, placebo-controlled trial. Trials. 2021 Dec;22(1):1-2.
Statistical methods	20a	Statistical methods for analysing primary and secondary outcomes. Reference to where other details of the statistical analysis plan can be found, if not in the protocol	Page No. 12<br>Line No. 2-6
	20b	Methods for any additional analyses (eg, subgroup and adjusted analyses)	Page No. 12<br>Line No. 9-14<br>Page No. 13<br>Line No. 1-12

	20c	Definition of analysis population relating to protocol non-adherence (eg, as randomised analysis), and any statistical methods to handle missing data (eg, multiple imputation)	Described in the below given study protocol at Page No.8 Sinha AP, Gupta SS, Poluru R, Raut AV, Arora NK, Pandey RM, Sahu AR, Bethou A, Sazawal S, Parida S, Bavdekar A. Evaluating the efficacy of a multistrain probiotic supplementation for prevention of neonatal sepsis in 0–2-month-old low birth weight infants in India—the "ProSPoNS" Study protocol for a phase III, multicentric, randomized, double-blind, placebo-controlled trial. Trials. 2021 Dec;22(1):1-2.
**Methods: Monitoring**			
Data monitoring	21a	Composition of data monitoring committee (DMC); summary of its role and reporting structure; statement of whether it is independent from the sponsor and competing interests; and reference to where further details about its charter can be found, if not in the protocol. Alternatively, an explanation of why a DMC is not needed	Described in the below given study protocol at Page No.3 Sinha AP, Gupta SS, Poluru R, Raut AV, Arora NK, Pandey RM, Sahu AR, Bethou A, Sazawal S, Parida S, Bavdekar A. Evaluating the efficacy of a multistrain probiotic supplementation for prevention of neonatal sepsis in 0–2-month-old low birth weight infants in India—the "ProSPoNS" Study protocol for a phase III, multicentric, randomized, double-blind, placebo-controlled trial. Trials. 2021 Dec;22(1):1-2.

For peer review only - http://bmjopen.bmj.com/site/about/guidelines.xhtml

	21b	Description of any interim analyses and stopping guidelines, including who will have access to these interim results and make the final decision to terminate the trial	Described in the below given study protocol at Page No.8 Sinha AP, Gupta SS, Poluru R, Raut AV, Arora NK, Pandey RM, Sahu AR, Bethou A, Sazawal S, Parida S, Bavdekar A. Evaluating the efficacy of a multistrain probiotic supplementation for prevention of neonatal sepsis in 0–2-month-old low birth weight infants in India—the "ProSPoNS" Study protocol for a phase III, multicentric, randomized, double-blind, placebo-controlled trial. Trials. 2021 Dec;22(1):1-2.
Harms	22	Plans for collecting, assessing, reporting, and managing solicited and spontaneously reported adverse events and other unintended effects of trial interventions or trial conduct	Page No. 8<br>Line No. 23-31
Auditing	23	Frequency and procedures for auditing trial conduct, if any, and whether the process will be independent from investigators and the sponsor	This being regulatory trial it is directly monitored by the DCGI
**Ethics and dissemination**			
Research ethics approval	24	Plans for seeking research ethics committee/institutional review board (REC/IRB) approval	Page No. 15<br>Line No. 10-14
Protocol amendments	25	Plans for communicating important protocol modifications (eg, changes to eligibility criteria, outcomes, analyses) to relevant parties (eg, investigators, REC/IRBs, trial participants, trial registries, journals, regulators)	We follow DCGI regulations

For peer review only - http://bmjopen.bmj.com/site/about/guidelines.xhtml

Consent or assent	26a	Who will obtain informed consent or assent from potential trial participants or authorised surrogates, and how (see Item 32)	Described in the below given study protocol at Page No.4 Sinha AP, Gupta SS, Poluru R, Raut AV, Arora NK, Pandey RM, Sahu AR, Bethou A, Sazawal S, Parida S, Bavdekar A. Evaluating the efficacy of a multistrain probiotic supplementation for prevention of neonatal sepsis in 0–2-month-old low birth weight infants in India—the "ProSPoNS" Study protocol for a phase III, multicentric, randomized, double-blind, placebo-controlled trial. Trials. 2021 Dec;22(1):1-2.
	26b	Additional consent provisions for collection and use of participant data and biological specimens in ancillary studies, if applicable	On 60th day of follow up additional informed consent was taken from the participants for Health economics study
Confidentiality	27	How personal information about potential and enrolled participants will be collected, shared, and maintained in order to protect confidentiality before, during, and after the trial	anonymize identifiable information
Declaration of interests	28	Financial and other competing interests for principal investigators for the overall trial and each study site	Page No. 16 Line No. 7-12
Access to data	29	Statement of who will have access to the final trial dataset, and disclosure of contractual agreements that limit such access for investigators	Page No. 16 Line No. 13
Ancillary and post-trial care	30	Provisions, if any, for ancillary and post-trial care, and for compensation to those who suffer harm from trial participation	As per GCP guidelines all these will be provided to participants on request of site Principal Investigator

For peer review only - http://bmjopen.bmj.com/site/about/guidelines.xhtml

Dissemination policy	31a	Plans for investigators and sponsor to communicate trial results to participants, healthcare professionals, the public, and other relevant groups (eg, via publication, reporting in results databases, or other data sharing arrangements), including any publication restrictions	Page No. 15 Line No. 12-13
	31b	Authorship eligibility guidelines and any intended use of professional writers	Page No. 15 Line No. 25-29 Page No.16 Line No.1-3
	31c	Plans, if any, for granting public access to the full protocol, participant-level dataset, and statistical code	Not yet planed
**Appendices**			
Informed consent materials	32	Model consent form and other related documentation given to participants and authorised surrogates	Uploaded as 'Supplemental Material' file
Biological specimens	33	Plans for collection, laboratory evaluation, and storage of biological specimens for genetic or molecular analysis in the current trial and for future use in ancillary studies, if applicable	As per existing SOP lab specific procedures

*It is strongly recommended that this checklist be read in conjunction with the SPIRIT 2013 Explanation & Elaboration for important clarification on the items. Amendments to the protocol should be tracked and dated. The SPIRIT checklist is copyrighted by the SPIRIT Group under the Creative Commons "Attribution-NonCommercial-NoDerivs 3.0 Unported" license.

For peer review only - http://bmjopen.bmj.com/site/about/guidelines.xhtml

BMJ Open

A Study Protocol for Economic Evaluation of Probiotic Intervention for Prevention of Neonatal Sepsis In 0-2 Month Old Low Birth Weight Infants In India - The ProSPoNS Trial

Journal:	*BMJ Open*
Manuscript ID	bmjopen-2022-068215.R2
Article Type:	Protocol
Date Submitted by the Author:	28-Feb-2023

Complete List of Authors:	sinha, anju; Indian Council of Medical Research, Reproductive and Child Health<br>Bahuguna, Pankaj; Post Graduate Institute of Medical Education and Research School of Public Health, Department of Community Medicine and School of Public Health<br>Gupta, Subodh; MGIMS, Sewagram, Community Medicine; MGIMS, Sewagram, Community Medicine<br>Kuruba, Yamini Priyanka; Indian Council of Medical Research, RBMCH<br>Poluru, Ramesh; The INCLEN Trust International,<br>Mathur, Apoorva; Indian Council of Medical Research, RBMCH<br>Raja, Dilip ; Indian Council of Medical Research, RBMCH<br>Raut, Abhishek; Mahatma Gandhi Institute of Medical Sciences, Department of Community Medicine<br>Mahajan, Kamaleshwar; Mahatma Gandhi Institute of Medical Sciences, Department of Community Medicine<br>Sudhakar, Rishikesh; INCLEN<br>Kulkarni, Bharati; National Institute of Nutrition, ;<br>Pandey, Ravindra Mohan ; All India Institute of Medical Sciences, Statistics<br>Arora, Narendra K. ; The INCLEN Trust International<br>Prinja, Shankar; Post Graduate Institute of Medical Education and Research, Community Medicine and School of Public Health; Post-graduate Institute of Medical Education and Research

**Primary Subject Heading**:	Paediatrics
Secondary Subject Heading:	Health economics
Keywords:	Neonatal intensive & critical care < INTENSIVE & CRITICAL CARE, HEALTH ECONOMICS, Paediatric intensive & critical care < INTENSIVE & CRITICAL CARE

SCHOLARONE™
Manuscripts

Title Page

A Study Protocol for Economic Evaluation of Probiotic Intervention for Prevention of Neonatal Sepsis In 0-2 Month Old Low Birth Weight Infants In India - The ProSPoNS Trial

Anju Pradhan Sinha*[1], Pankaj Bahuguna[2], Subodh S. Gupta[3], Kuruba Yamini Priyanka[1], Ramesh Poluru[4], Apoorva Mathur[1], Dilip Raja Koppula[1], Abhishek V. Raut[3], Kamaleshwar Mahajan[3], Rishikesh Sudhakar[4], Bharati Kulkarni[1], Ravindra Mohan Pandey[5], Narendra Kumar Arora[4], Shankar Prinja[2]

1. Division of Reproductive, Child Health & Nutrition, Indian Council of Medical Research, New Delhi
2. Department of Community Medicine & School of Public Health, Post Graduate Institute of Medical Education and Research, Chandigarh, India
3. Department of Community Medicine, Mahatma Gandhi Institute of Medical Sciences, Wardha, India
4. The INCLEN Trust International, New Delhi, India
5. Ex. Head of the Department of Biostatistics, All Indian Institute of Medical Science, New Delhi, India

*** Corresponding author details:**

Anju Pradhan Sinha, Indian Council of Medical Research, Reproductive and Child Health, Ansari Nagar, New Delhi-110029, Email ID: apradhandr@gmail.com, Mobile Number: 09811422241

Keywords: Neonatal Sepsis, Low Birth Weight, Economic Evaluation, Probiotics, Disability adjusted life year

Word count (excluding title page, abstract, references, figures and tables):3951 words

Number of References: 19

Abstract count: 285 words

For peer review only

Abstract

Introduction: The ProSPoNS trial is a multicentre, double-blind, placebo-controlled trial to evaluate the role of probiotics in prevention of neonatal sepsis. The present protocol describes the data and methodology for the cost utility of the probiotic intervention alongside the controlled trial.

Methods and analysis: A societal perspective will be adopted in the economic evaluation. Direct medical and non-medical costs associated with neonatal sepsis and its treatment would be ascertained in both the intervention and the control arm. Intervention costs will be facilitated through primary data collection and program budgetary records. Treatment cost for neonatal sepsis and associated conditions will be accessed from Indian national costing database estimating healthcare system costs (NHSCD). A cost –utility design will be employed with outcome as incremental cost per disability-adjusted life year (DALY) averted. Considering a time-horizon of 6 months, trial estimates will be extrapolated to model the cost and consequences among high-risk neonatal population in India. A discount rate of 3% will be used. Impact of uncertainties present in analysis will be addressed through both deterministic and probabilistic sensitivity analysis.

Ethics and Dissemination: Has been obtained from EC of the six participating sites (MGIMS Wardha, KEM Pune, JIPMER Pudduchery, AIPH, Bhubaneswar, LHMC New Delhi, SMC Meerut) as well as from the ERC of LSTM, UK. A peer reviewed article will be published after completion of the study. Findings will be disseminated to the community of the study sites, with academic bodies and policy makers.

Registration: The protocol has been approved by the regulatory authority (Central Drugs Standards Control Organisation; CDSCO) in India (CT-NOC No. CT/NOC/17/2019 dated 01 March 2019). The ProSPoNS trial is registered at the Clinical Trial Registry of India (CTRI). CTRI/2019/05/019197. Registered on 16 May 2019

Strengths:

- This would be the first study evaluating cost effectiveness of using probiotic intervention among low birth weight newborn infants in India.

For peer review only

- This study would inform incremental costs per DALY averted with use of probiotics which will be key for prioritising resource allocation in view of other competing demands for child health, therefore crucial for public policy decisions.
- Data from the RCT would be complimented with the use of a model-based approach to explore scenarios beyond the trial data.

Limitations:

- Generalisability and external validity of findings from economic evaluation concurrently performed with the main study may not represent the same treatment effects and costs as in routine clinical practice.

Introduction

There is progress towards attaining the 3rd Sustainable Development Goal (SDG) of under-five (U5) mortality of 25 per 1000 live births by 2030, in India. Neonatal mortality rate is the most important contributing factor of the U5 mortality [1, 2]. Further decline in neonatal mortality may require newer interventions for prevention or treatment of neonatal infections like meningitis, pneumonia, septicaemia that result in more than a quarter of the 1 million neonatal deaths every year in India [2]. The ProSPoNS trial [3] proposes to evaluate the role of probiotics in prevention of neonatal infections (sepsis) among the vulnerable group of low birth weight infants contributing the highest rates of morbidity and mortality. A larger sample size and precise outcome measures differentiate this trial from the earlier conducted pilot study that showcased a 21% non-significant decline in morbidity due to neonatal sepsis in the intervention arm. Considering the 30% prevalence of low-birth weight (LBW) in India and 30 % mortality due to neonatal sepsis, even a small decline in the incidence of sepsis would translate to a large number of new born lives saved. [3]

If the intervention is proven to be efficacious, it would be important to know whether it is also a cost effective intervention. There are few examples in the literature where economic evaluations have been conducted on other effective interventions to prevent neonatal sepsis.[4,5] It cannot be overemphasized that along with clinical effectiveness of interventions, an estimate of economic effectiveness either in terms of costs or utilities (DALYs and QALYs) is required to make correct choices about their inclusion in public health programs. This is of immense importance in presence of multiple competitive options where prioritization is needed.

For peer review only

Ranjeva et al. [6] have quantified the scope of the public health and economic burden of neonatal sepsis in Sub-Saharan Africa. However, evidence on the health economics aspect of neonatal sepsis prevention effect of probiotics has not been ascertained so far. We propose to evaluate the cost utility of probiotic intervention for prevention of neonatal sepsis within the ProSPoNS trial,

Background

Study Setting & Study population

ProSPoNS trial is a Phase III multicentre randomized, double-blind placebo-controlled trial, being conducted at six study sites across India. It is a global Health trial funded by the UK Research and Innovation (UKRI).

The Study is ongoing at 6 sites across India: Jawaharlal Institute of Postgraduate Medical Education and Research (JIPMER), Puducherry, Mahatma Gandhi Institute of Medical Sciences (MGIMS), Wardha, King Edward Memorial Hospital (KEM Hospital), Pune, Asian Institute of Public Health (AIPH), Bhubaneswar (with recruitments from SCB Medical College and Hospital, Cuttack), Subharti Medical College (SMC) and Lala Lajpat Rai Memorial Medical (LLRM) College District women hospital, Meerut and Lady Hardinge Medical College (LHMC), Kalawati Saran Children's Hospital (KSCH), Delhi. The primary objective is to estimate a 30% reduction in the incidence of neonatal sepsis in the intervention arm with daily supplementation of 1ml (10 billion CFU/ml) of Vivomixx drops over a period of 30 days in 0-2-month-old LBW infants. 6144 Low birth weight (between 1500-2500 g) infants would be recruited to the study from the 6 study sites located in Bhubaneswar, Meerut, New Delhi, Puducherry, Pune, and Sewagram, between ages 3-7 days of life.

Theory of change

More than one-third of the estimated four million neonatal deaths around the world each year are caused by severe infections, and a quarter - around one million deaths - is due to neonatal sepsis/pneumonia alone. Panigrahi et al in their study which was conducted in the year 2017 reported that the Culture confirmed incidence of sepsis as 6.7/1000 births with 51% Gram negatives (Klebsiella predominating) and 26% Gram positives (mostly Staphylococcus aureus). The Sustainable Development Goal for child survival cannot be achieved without substantial reductions in infection-specific neonatal mortality [1]. Due to non-specific signs and symptoms, sepsis is difficult to diagnose. The chances of recovery and survival depend on the correct diagnosis, referral, reaching a facility and adequate treatment by appropriately-trained health workers and high-quality services.

For peer review only

Low Birth Weight is one of the important indirect causes of deaths in neonates accounting for 40-80% of neonatal deaths worldwide [2]. LBW neonates are immune compromised and manifest poor cognitive functions [7]. Since LBW infants are more prone to infections, neonatal mortality and morbidity may be decreased by preventing neonatal infection.

Antibiotic therapy is the mainstay of current therapeutic management of neonatal infections. For serious infections in new borns and young infants, there is a lack of guidelines on rational antibiotic use [8]. In the absence of rapid diagnostics, non-judicious use of antibiotics contributes to the rising antimicrobial resistance. Antibiotic overuse is linked with disturbing the gut ecosystem. The problem of drug resistance outweighs the fast pace of newer generation antibiotic production.

Currently, there are no preventive interventions available for neonatal sepsis other than general measures of hand washing, exclusive breastfeeding, etc. However evaluating immunotherapy with immune globulin, probiotics, myeloid colony-stimulating factors, glutamine supplementation, recombinant human protein C and lactoferrin are being evaluated as adjuvants for the prevention of neonatal sepsis [9]. Probiotics are non-pathogenic microbes which are capable of exerting positive health effects in the host body through various mechanisms [10] [11]. It acts by suppressing the growth of potential pathogens and their epithelial attachment. It also works by secreting anti-microbial substances or by stimulating host expression of protective molecules. Besides, it can produce a barrier against the harmful pathogens at higher levels. It stimulates the process of immunosuppressive agent's production in the host which suppress the inflammatory responses or accelerate the immunologic mechanism that clears the infection. Probiotics stimulate normal microbe-microbe and host-microbe interactions positively, thereby enhance the protection provided by commensal flora through competitive interactions, pathogenic antagonism, and antimicrobial factors. We hypothesize that immunomodulation/ immunopotentiation with probiotics may prove to be an alternative for the prevention of neonatal sepsis. **Figure 1** depicts the conceptual framework to prevent sepsis episodes among 0-2 months neonatal population with use of probiotics.

Research Question:

Is probiotic (vivomax) use as prophylaxis a cost-effective strategy for preventing sepsis among 0-2 month old infant population in India?

Objectives: To assess the cost utility of using probiotics as prophylaxis for sepsis among LBW neonates of age 0 to 2 months.

For peer review only - http://bmjopen.bmj.com/site/about/guidelines.xhtml

Study Methodology

A cost utility analysis is proposed to be conducted alongside the multicentre RCT. Randomized controlled trials (RCT) are considered the 'gold standard' as they produce a reliable the best evidence of treatment effect. The economic evaluation alongside RCT provides opportunity to collect data both on disease-specific disability and resource use. Start date is 15th November 2022 and the anticipated completion date is December 2023

Model Overview

General Description:

This protocol describes the methodology for economic evaluation within a phase III RCT – the ProSPoNS trial. The details are described in the following sections.

Decision Model

In view of the shortcomings of treatment effect measure coming from a single RCT, we also propose using a model-based approach which will allow synthesizing information from all sources for a health technology enabling us to explore scenarios not explicitly found in the trial data. Moreover, the modelling approach would enable to estimate the long-term benefits of intervention incorporating quality of life measures. Furthermore, it allows accounting for uncertainties present in assumptions and data used for economic evaluation, which has implications for policy question under consideration. The clinical outcomes from trial would be sepsis episodes averted as an effect of probiotics use in the intervention arm. Quality of life (QUALY) is a preferred utility measure for economic analysis, however there is no EQ-5D version for neonates. Therefore, in order to assess the long-term effects, we propose to use 'Disability adjusted life years' (DALY) as the measure of health outcome which accounts for both disability and quality of life. A decision-analytic model following a systematic approach in bringing together evidence on clinical effectiveness, health-related disability of life and costs associated with the probiotic intervention as compared to placebo will be used with the aim to provide information on whether the reduction in neonatal sepsis incidence justifies the incremental costs. **Figure 2** depicts the decision model for the proposed study.

Outcome measures: Primary outcome measure for economic evaluation (within-trial CUA) would be sepsis episodes and related deaths averted in the intervention arm as compared to the placebo arm, which translates to gain in life years and DALYs averted as long-term measure.

For peer review only - http://bmjopen.bmj.com/site/about/guidelines.xhtml

Data requirements: Data requirements for economic evaluation is tabulated in Table-1.

Table- 1: Data requirements for economic evaluation:

**Data Type**	**Significance for the Model**	**Source of Data**
Demographic	- Estimate the baseline parameters<br>- Estimate target population	Primary data collection; Census of India
Treatment effects & Incidence of Adverse Events	- Efficacy and safety parameters<br>- Cost estimation<br>- Outcome assessment	RCT Database
Background Mortality data	- Estimate natural mortality in the target population	SRS, Published literature
Utilities	- Estimate DALY as long-term outcome measure	Global Burden of Diseases estimates (2011)
Resource use and price data	- Cost estimation	Primary data collection; NHSC-Prinja et al
Epidemiological data	- Estimate baseline risk of clinical events	Published literature; Primary data collection (Control arm of the RCT)

RCT- Randomised Control Trial, SRS- Sample Registration System, NHSCD- National Health Systems Costing Database

Utilities data would allow incorporating the relative impact of a health technology (i.e. probiotics) on the neonatal health outcomes in terms of disability burden. This will be estimated as DALYs averted. Utility data would be obtained from Global disease burden estimates 2017. Cost data are important drivers of cost-effectiveness, as they quantify the resource consumption associated with use of health technologies.

Decision-analytic model would be populated with cost data from the societal perspective: direct medical and non-medical cost borne by patients; and direct medical cost borne by the health care system. A national health system costing database (NHSCD) to estimate health care system's costs in India has been developed. [12] Data on patient's out-of-pocket (OOP) expenses would be collected during the conduct of the RCT as primary data.

A health economic evaluation using cost- utility analysis (CUA) design will be conducted within the ProSPoNS trial. The results would provide the incremental costs and health gains of the probiotic

For peer review only

intervention in absence of any prevention modality being implemented or available. Decision-analytical modelling using the trial results would be extrapolated to the target population and time horizon to inform policy on the potential for incremental costs offsets attributable to prevention of morbidity with the proposed intervention. The CUA will use the present results as cost per case of neonatal sepsis prevented. CUA will present the results as cost per disability-adjusted life year averted. Intervention costs and effects will be extrapolated to the Indian neonatal population. Modelling will be used for the target population, time horizon, and the decision context to provide much required information for the policymakers. It would guide further decision for up scaling the intervention, if it is found to be efficacious. If the incremental costs of the intervention can be offset by the gains in terms of effectiveness (i.e. DALYs) it may be presented for induction within the ambit of existing governmental program. Modelled CUA results can be presented as health - adjusted life years saved and savings of the health care system by disease prevention. We will calculate the incremental cost-effectiveness ratios as the difference in the costs between the intervention and the comparator divided by the difference in health outcomes.

Effectiveness assessment:

Prevention of severe sepsis in the early neonates will prevent the long-term morbidity like neurodevelopmental disability risk associated with sepsis [13] and reduce mortality due to pneumonia, meningitis, and neonatal sepsis and hospital admissions due to any infectious diseases [14].

We propose to use the short time horizon to assess the sepsis averted as a result of probiotics supplementation for one month and model the change in DALYs in the intervention group as a long-term measure.

Study details:

Total of 6144 (N) new-born babies with LBW would be screened for eligibility and randomly assigned to probiotic (n=3072) or placebo (n=3072) groups and administered 1 ml of the probiotic suspension daily for 30 days. The study would use IWRS for the purpose of stratified randomization by birth weight, sex and study site. Trained field workers will make home visits daily for first 7 days and thereafter thrice in a week till day-30 of the follow up period to ensure and monitor adherence to the intervention and collect morbidity information. After the 30-day intervention period, home visitation will continue at weekly interval till day-60 of life. During home visitation as per schedule trained fieldworkers would screen infants for symptoms/ signs of PSBI/ Clinical illness/ Clinical severe infection. To record the AE/SAE events (if any), continuous monitoring and reporting of

For peer review only

ongoing medical conditions, signs symptoms will be conducted by field worker during their scheduled follow-up home visits using software on handheld device during the two-months period of follow up. Presence of any danger signs would be evaluated, and need for referral would be assessed: If a child is found to have any symptoms/ signs from the list, the field worker will accompany the infant to facility for examination by study physician (referral). The study physician would examine the infant again for symptoms/ signs of PSBI/ Clinical illness/ Clinical severe infection and confirm the diagnosis. Investigations such as blood culture, sepsis screen would be performed for suspected cases of neonatal sepsis by study physician. If required the infant would be hospitalized for treatment. Data on outcomes, lab investigation and treatment during clinical management of the sick new-born will be collected and serious adverse events will be reported as per regulatory guidelines. Blood culture isolates will be shipped by site to microbiology lab at All India Institute of Medical Sciences (AIIMS), New Delhi for quality assurance purpose.

The study will be implemented according to MRC guidelines for management of global health trials and will be governed by a Trial Steering Committee and a Data Management Committee.

Primary outcomes:

1. Sepsis: defined as one or more clinical symptoms suggestive of sepsis with a microbial isolate on blood culture; or a neonate with sterile blood culture with at least 2 sepsis screen markers being abnormal (Absolute neutrophil count<1500/mm3; TLC<5000/mm3; Immature to total neutrophil ratio > 0.2; Immature to total neutrophil ratio > 0.2;CRP >1.2 mg/dl, ESR>15 mm).

2. Possible Serious Bacterial Infection (PSBI): One or more clinical signs like fever (temperature ≥ 38 °C), low body temperature (< 35.5 °C), not feeding well, convulsions, fast breathing (60 breaths per minute or more), severe chest in-drawing, movement only when stimulated or no movement at all in infants less than 7 days old confirmed by study physician.

Secondary outcomes:

1. Clinical severe infection: One or more clinical signs like severe chest in-drawing, not feeding well, fever (temperature ≥ 38 °C), the movement only when stimulated as confirmed by the study physician, low body temperature (< 35.5 °C).

2. Critical Illness: One or more of clinical signs like unable to feed at all, unable to cry, no movement on stimulation, bulging fontanelle, cyanosis as confirmed by the study physician and convulsions. The flow chart below (Figure 1) depicts the study design and the study processes that will be

For peer review only

followed during the implementation of the proposed research work (Diagram, to be finalised after finalisation of methods). Primary and secondary outcomes would be compared between the two study arms to assess clinical effectiveness of probiotic intervention.

Costing

In the current study, cost of Vivomix (intervention), cost of routine home-based postnatal care (HBPNC), cost of illness management, drug costs, vitamin/mineral supplement costs, laboratory investigation charges, and doctor consultation, bed charges are categorized as direct medical costs and the informal payments and travel are categorized as the direct non-medical costs. Loss of wages of parents and other caregivers due to absenteeism from work and is categorized as indirect costs. Neonatal sepsis episodes are treated in in-patient setting; therefore respondents (parents and caregivers) will be interviewed to collect information on days they were absent from work due to hospital stay for treatment. After hospital discharge, follow up with parents and caregivers to assess any effect on their productivity at work is beyond the scope of data collection in the present study. All costs related data would be collected from all six study sites in a representative manner.

Sample size (Costing): We calculated the sample size to estimate the mean out-of-pocket (OOP) expenditures among the neonates with illness (here PSBI). A power of 80% and 5% level of significance was assumed for sample size calculation. The baseline estimate for OOP among neonates with PSBI was accessed from an Indian study. [1] Assuming a mean OOP of □2003 (Std deviation: □3517) for neonates with PSBI and an error margin of 7.5%, the minimum sample size computed to estimate the OOP was 1998. In view of high variability in the OOP data, the error margin of 7.5% was considered appropriate for sample size calculation. The data on OOP will be captured for a sub-sample i.e. 1998 neonates, out of total sample recruited in the study. At each of the six subjects recruitment sites data would be collected on 333 subjects for this sub-study. The OOP data will be collected irrespective of status of neonates belonging to two study arms. The formula used for sample size calculation is given below:

$$n = \frac{Z^2_{1-a/2}\, s^2}{d^2}$$

Where; n= minimum estimated sample size

For peer review only

Z^2_{1-a}= table value from standard normal distribution
s^2= standard deviation of outcome variable in study population
d^2 = error margin (or absolute precision)

Data collection & Estimation of costs: Data collection for the current study would be completed in one year duration from September 2022 to August 2023 (to cover any variation with seasonality). Data on out-of-pocket expenditures (OOP) will be collected on a sub-sample of 333 subjects drawn from the total trial population in a representative manner.

Socio- demographic data on education, occupation of parents of enrolled children is being collected on study forms. Parents would be asked about any illness during the study period and any expenditures on account of care seeking, visits to facilities, hospitalization and treatment. Direct medical and non-medical costs due to neonatal sepsis would be ascertained in all 333 subjects at the six study sites. Data on OOP expenditure in public or private health facilities would be elicited.

Intervention cost would be facilitated through primary data collection and the program budgetary records. The cost of treatment for neonatal sepsis and related diseases at secondary and tertiary level will be ascertained from the NHSCD[15,16] and published literature in Indian context.[12] These sources provides estimates on cost per in-patient (IP) admission, cost of intensive care unit (ICU) admission, cost per out-patient (OP) consultation accounting for resources consumed for service provision such as medical (drugs, diagnostics, procedural, hospital charges) and non-medical (transportation, boarding & lodging). In addition, cost related to IEC material, monitoring & supervision and other administrative costs will also be estimated. Assumptions made in the study protocol are provided in Table 2.

Table 2: Key methods and assumptions for economic evaluation:

Component	Consideration/Approach	Justification
Intervention	- Vivomixx (8 strain probiotics) + Sepsis management<br>- Dose: 1ml/day (10 billion CFU/ml) for 30 days<br>- Intervention delivery: Under the supervision of study staff (field worker)	Effectiveness of Vivomixx for preventing neonatal sepsis is being assessed under trial
Comparator	- Placebo	- Effectiveness of Vivomixx is being assessed under trial against Placebo<br>- Use of antibiotic is routine practice for sepsis management.

For peer review only

Target Population	- High-risk LBW neonates (Both pre-term and small for-date babies)	Most vulnerable high-risk population with high morbidity & mortality
Objective	- To assess the incremental cost per DALY averted with use of probiotics for prevention of neonatal sepsis compared the placebo	Besides effectiveness, evidence on cost-effectiveness is mandatory for adoption of treatment/intervention under public policy
Target Audience/Policy Use	- National Health Mission (NHM)<br>- Any other	This intervention is to be implemented under RMNCH program
Method	- Both model and trial based	- Short –term outcome estimates will be used for trial<br>-Use of model-based CEA will enable us to assess long-term outcomes (and sub-group analysis)
Perspective	- Societal perspective	OOP burden associated with sepsis management among LBW neonates
Analytic Horizon	- 6 months	To model long-term outcomes (DALYs)
Costs	- Health system costs (Vivomixx cost; Cost of routine HBPNC care; cost of illness management etc.)<br>- OOP expenditures (Doctor's consultation; bed charges; Drug costs; Vitamin/Mineral supplement costs; Investigations; Procedures; Informal payments; Travel cost etc.	Both health system and patients' costs as per societal perspective considered for CEA
Outcomes	- Sepsis episodes and deaths averted<br>- DALYs<br>- Hospitalization averted<br>- Life years gained	In line with study design (cost-effectiveness/cost-utility analysis)
Discount rate	- 3%	As per Indian reference case

1

For peer review only

Total cost will be comprised of cost of intervention, cost of management of sepsis and associated complications and patient costs as OOP. Similarly, total cost in the control arm will be estimated in the absence of intervention. Annual discount rate of 3% will be used wherever applicable. In the present study both model based approach as well as trial data would be used. Data management and analysis will be done using MS-Excel.

Other Analyses: Uncertainty analyses, Budget impact analysis, Stakeholder analysis would be included.

Uncertainty Analysis: Both deterministic and probabilistic sensitivity analysis (PSA) will be undertaken to address the effect of uncertainties present in analysis. For PSA, all the parameter values will be varied within a plausible range. Based on nature of parameter, an appropriate probabilistic distribution will be assigned such as beta distribution for risk and utility parameters, gamma distribution for cost parameters, uniform distribution for demographic parameters etc. The results under PSA will be simulated 999 times to report a 95% confidence interval for ICERs.

Budget Impact Analysis: A Budget impact analysis is proposed to be conducted to estimate the financial consequences of increasing uptake of probiotic supplementation for LBW infants in the neonatal care under NHM, over the near future (3 to 5 years). Data regarding the target population of LBW infants will be available by extrapolating the data from the RCT, as will be other epidemiological inputs to populate the budget-impact model. At present there are no alternative prevention modalities for neonatal sepsis present (other than hand hygiene and exclusive breastfeeding). Therefore, assumption would be made regarding the uptake of probiotic supplementation (based on the results of the study).

Stakeholder analysis: A range of potential stakeholders will be consulted, involving experts such as neonatologists/paediatricians, academics, policy makers, program managers, industry, patient group representatives etc. Multiple consultations with stakeholders will be done during the conduct of study as well as dissemination of study results.

Patient and Public Involvement: Patients or caregivers or members of public were not involved in the development of this protocol

For peer review only

Discussion

The ProSPoNS trial is evaluating the role of probiotics intervention in prevention of neonatal infections, a major cause for neonatal mortality. To date no prevention modalities other than hand hygiene and exclusive breastfeeding have been successfully incorporated in programs for prevention of neonatal infections. Panigrahi el al (2017) established benefit of Lactobacillus planitarum with fructooligosaccharides intervention in prevention of sepsis and related deaths in new-borns; however, the study did not assess the cost effectiveness of the intervention. There is no study in the literature evaluating the cost-effectiveness of this novel disease prevention technology. We have proposed such evaluation alongside the ProSPoNS trial.

A RCT design, considered a gold standard for such evaluations is being used in the trial. However, there are disadvantages of using RCT data quoted in the literature. Data from a single RCT may not be adequate for estimation of cost utility as RCTs mainly focus on a single intervention and have small follow up period, thus, lack to provide evidence on effectiveness of multiple alternatives and situations beyond the trial conditions. The treatment effect measured in a single study may be relevant for a particular setting but has limited generalizability of results in populations other than the one studied. [17, 18, 19]. We propose using a modelling approach to overcome the above mentioned shortcomings of RCT data.

There are a few examples of estimation of cost-effectiveness of disease preventive technologies in the neonatal population. A study conducted in Sub-Saharan Africa estimated that neonatal sepsis resulted in 5.29-8.73 DALYs lost annually accounting for an economic burden from $10 billion to $469 billion (per annum). It was recommended that a strategic plan for the treatment and prevention of neonatal sepsis may reduce this burden.[6] A cost-effective analysis conducted in Ghana reported that alcohol-based hand rub (ABH) effectively reduced the patient cost of neonatal bloodstream infection (BSI), Hospital cost associated with BSI, length of hospital stay, and deaths due to BSI by 41.7%, 48.5%, 50% and 73% respectively. It was also estimated that ABH reduced the incidence of hospital-acquired neonatal infections by 16%.[4]. Current study would estimate the cost-effectiveness of a novel neonatal sepsis prevention modality.

In considering cost-effectiveness, measures on treatment effect over longer time horizon than RCTs are often required. Therefore we have proposed a modelling approach in addition to the use of primary data coming from the ongoing RCT. This approach would use and provide a wider range of assumptions/parameters from multiple data sources and evaluate the long term utility of the probiotic intervention in disease prevention.

For peer review only

Expected outcomes of study and policy implications: The health economics evaluation would generate data on cost involved with the new intervention as compared to the control arm with uncertainty range, Incremental costs, and health outcomes for both study arms with uncertainty range, ICER, sensitivity and threshold analyses. It would be helpful for the policy maker to understand the cost utility of the probiotic intervention in presence of other competing priorities.

Strengths:

- This would be the first study evaluating cost effectiveness of using probiotic intervention among low birth weight newborn infants in India.
- This study would inform incremental costs per DALY averted with use of probiotics which will be key for prioritising resource allocation in view of other competing demands for child health, therefore crucial for public policy decisions.
- Data from the RCT would be complimented with the use of a model-based approach to explore scenarios beyond the trial data.

Challenges:

- Generalisability and external validity of findings from economic evaluation concurrently performed with the main study may not represent the same treatment effects and costs as in routine clinical practice.

Registration: The protocol has been approved by the regulatory authority (Central Drugs Standards Control Organisation; CDSCO) in India (CT-NOC No. CT/NOC/17/2019 dated 01 March 2019). The ProSPoNS trial is registered at the Clinical Trial Registry of India (CTRI). CTRI/2019/05/019197. Registered on 16 May 2019

For peer review only

Ethics and Dissemination: Approval has been obtained from EC of each of the six participating sites (MGIMS Wardha, KEM Pune, JIPMER Pudduchery, AIPH, Bhubaneswar, LHMC New Delhi, SMC Meerut) as well as from the ERC of LSTM, UK. A peer reviewed article will be published after completion of the study. Findings will be disseminated to in the community of the study sites, with academic bodies and policy makers.

This manuscript is in compliance with the SPIRIT checklist (Research Checklist file)

Author affiliations: Anju Pradhan Sinha[1], Pankaj Bahuguna[2], Subodh S. Gupta[3], Kuruba Yamini Priyanka[1], Ramesh Poluru[4], Apoorva Mathur[1], Dilip Raja Koppula[1], Abhishek V. Raut[3], Kamaleshwar Mahajan[3], Rishikesh Sudhakar[4], Bharati Kulkarni[1], Ravindra Mohan Pandey[5], Narendra Kumar Arora[4], Shankar Prinja[2] & the ProSPoNS Trial group of investigators.

6. Indian Council of Medical Research, New Delhi
7. Post Graduate Institute of Medical Education and Research, Chandigarh
8. Mahatma Gandhi Institute of Medical Sciences, Wardha
9. INCLEN Trust International, New Delhi
10. All Indian Institute of Medical Science, New Delhi

Author Contributions: APS, SSG, NKA, AVR, RP conceptualized and developed the research question. PB, SP, KYP, AM, DRK developed the methodology, tools for data collections and framed the analysis plan. BK, SSG, APS, KM, RS, RMP, NKA involved in study protocol wrting, review and editing. APS, PB, KYP developed the original draft of manuscript.

List of Authors: ProSPoNS Trial group of investigators Ashish Bavdekar, Sunil Sazawal, Sushma Nangia, Sailaja Nandan Parida, B. Adhsivam.

Funding: The study is funded by the Department of Health and Social Care (DHSC), the Foreign, Commonwealth & Development Office (FCDO), the Medical Research Council (MRC) and Wellcome. Award/grant number is MR/S004912/1. The funding body has no role in the designing of the study and collection, analysis, and interpretation of data and in writing the manuscript.

Conflicts of Interests: The Authors declare that they have no conflicts of Interests.

For peer review only

Data Statement: We are in compliance with BMJ's Tier 2 data policy. We confirm that we are following the ICMJE's guidelines. Deidentified participant data will be shared with individuals, research agencies; funding agencies and systematic reviewers for network Meta analysis upon request to the corresponding author.

Acknowledgements: We would like to thank the Indian Council of Medical Research (ICMR) for the permission to conduct the study and the UK Research and Innovation, Medical Research Council, UK, for funding the trial under the Global Health Trials [JGHT] scheme 8, jointly funded by the DFID/NIHR/MRC/Wellcome Trust. We thank Dr. Soumya Swaminathan, Ex-Director General of the Indian Council of Medical Research (ICMR), New Delhi and late Prof. M.K. Bhan, for their motivation, encouragement and support with this study. The authors also thank M/s Next Gen Pharma India Pvt. Ltd., India, for being the trial partners (supply of the Active Pharmaceutical Ingredient (API)) and M/s Cadila Healthcare Ltd., India, for agreeing to manufacture and supply the finished formulation: probiotic drops and corresponding placebo used in the study. The authors especially thank the Liverpool School of Tropical Medicine Research Ethics Committee (LSTM REC), IEC of each participating site (participant recruitment site and laboratory site), the Data Monitoring Committee, and the Trial Steering Committee (TSC) for their review and inputs.

For peer review only

References:

1. India Newborn Action Plan (INAP): National Health Mission [Internet]. [Cited 2021 Mar 10]. Available from: https://nhm.gov.in/index4.php?lang=1&level=0&linkid=153&lid=174 https://www.newbornwhocc.org/INAP_Final.pdf.
2. Sachs JD. From millennium development goals to sustainable development goals. Lancet. 2012; 379(9832):2206–11. https://doi.org/10.1016/S0140-673 6(12)60685-0.
3. Sinha AP, Gupta SS, Poluru R, Raut AV, Arora NK, Pandey RM, Sahu AR, Bethou A, Sazawal S, Parida S, Bavdekar A. Evaluating the efficacy of a multistrain probiotic supplementation for prevention of neonatal sepsis in 0–2-month-old low birth weight infants in India—the "ProSPoNS" Study protocol for a phase III, multicentre, randomized, double-blind, placebo-controlled trial. Trials. 2021 Dec; 22(1):1-2.
4. Fenny AP, Otieku E, Labi KA-K, Asante FA, Enemark U (2022) Cost-effectiveness analysis of alcohol handrub for the prevention of neonatal bloodstream infections: Evidence from HAI-Ghana study. PLoS ONE 17(3): e0264905.
5. Nice Clinical Guideline 2012 on Antibiotic for early-onset neonatal infection.
6. Ranjeva SL, Warf BC, Schiff SJ. Economic burden of neonatal sepsis in sub- Saharan Africa. BMJ Glob Health2018; **3:e000347**. doi:10.1136/ bmjgh-2017-000347
7. Allen L and Gillespie S. What works? A review of the efficacy and effectiveness of nutrition interventions. ACC/SCN Nutrition Policy Paper No 19, ADB Nutrition and Development series No 5, 2001: 5-22.
8. Lin HC, Su BH, Chen AC, et al. Oral probiotics reduce the incidence and severity of necrotizing enterocolitis in very low birth weight infants. Paediatrics 2005; 115(1): 1-4
9. Wolkowiez MC, Benjamin DK, Capparelli E. Immunotherapy in neonatal sepsis: Advances in treatment and prophylaxis. Curr Opin Pediatr 2009; 21(2):177-181.
10. MarteanP, Seksik P, Jian R Probiotics and intestinal health effects: a clinical perspective. Br J. Nutr;2002,Sep; 88 Suppl 1:S51-7
11. R. Balfour Sartor, Probiotic therapy of intestinal inflammation and infections. Current opinion in Gastroenterology 2005, 21:44-50
12. Prinja S, Brar S, Singh MP, et al. Process evaluation of health system costing - Experience from CHSI study in India. PLoS One. 2020; 15(5):e0232873. Published 2020 May 13. doi:10.1371/journal.pone.0232873

13. Cai S, Thompson DK, Anderson PJ, Yang JY. Short- and Long-Term Neurodevelopmental Outcomes of Very Preterm Infants with Neonatal Sepsis: A Systematic Review and Meta-Analysis. Children 2019, 6, 131; doi:10.3390/children6120131
14. Wiens MO, Kissoon N, Holsti L. Challenges in pediatric post-sepsis care in resource limited settings: a narrative review. Transl Pediatr 2021; 10(10):2666-2677.
15. Prinja S, Chauhan AS, Rajsekhar K, Downey L, Bahuguna P, Sachin O, Guinness L. Addressing the cost data gap for universal healthcare coverage in India: a call to action. Value in Health Regional Issues. 2020 May 1;21:226-9.
16. National Cost database.National Health System Cost Database for India. Access on https://www.healtheconomics.pgisph.in/costing_web/
17. Briggs A, Sculpher M, Claxton K. Decision modelling for health economic evaluation. Oup Oxford; 2006 Aug 18.
18. Drummond MF, Sculpher MJ, Claxton K, Stoddart GL, Torrance GW. Methods for the economic evaluation of health care programmes. Oxford university press; 2015 Sep 25.
19. Glick HA, Doshi JA, Sonnad SS, Polsky D. Economic evaluation in clinical trials. OUP Oxford; 2014 Oct 2.

Figure legends:

Fig.1 Conceptual framework to prevent neonatal sepsis using probiotics.

Fig.2. Decision tree for cost effectiveness of using probiotics as prophylaxis for sepsis among neonates of age 0 to 2 months.

For peer review only

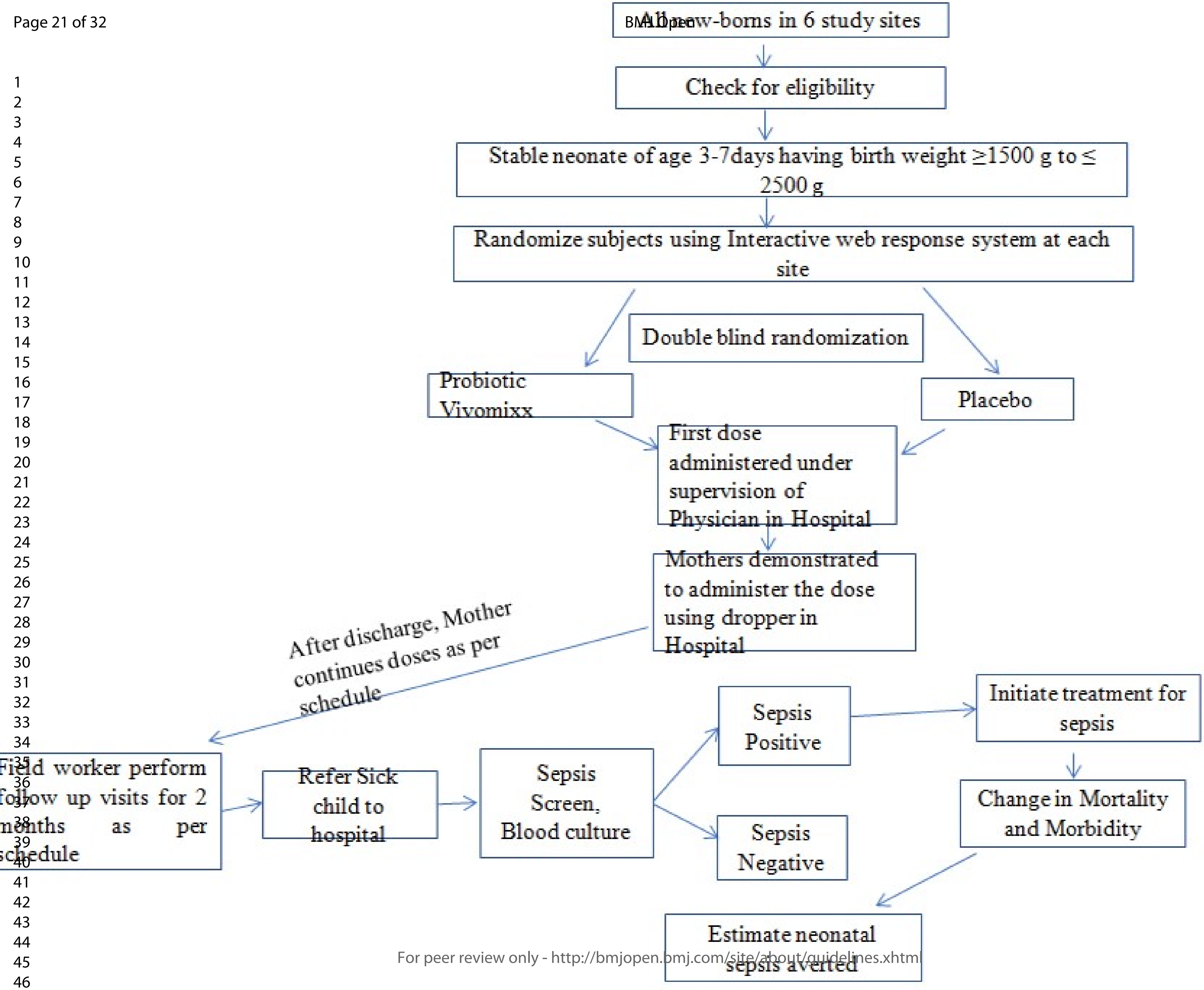
All new-borns in 6 study sites
Check for eligibility
Stable neonate of age 3-7days having birth weight ≥1500 g to ≤ 2500 g
Randomize subjects using Interactive web response system at each site
Double blind randomization
Probiotic Vivomixx
Placebo
First dose administered under supervision of Physician in Hospital
Mothers demonstrated to administer the dose using dropper in Hospital
After discharge, Mother continues doses as per schedule
Field worker perform follow up visits for 2 months as per schedule
Refer Sick child to hospital
Sepsis Screen, Blood culture
Sepsis Positive
Sepsis Negative
Initiate treatment for sepsis
Change in Mortality and Morbidity
Estimate neonatal sepsis averted

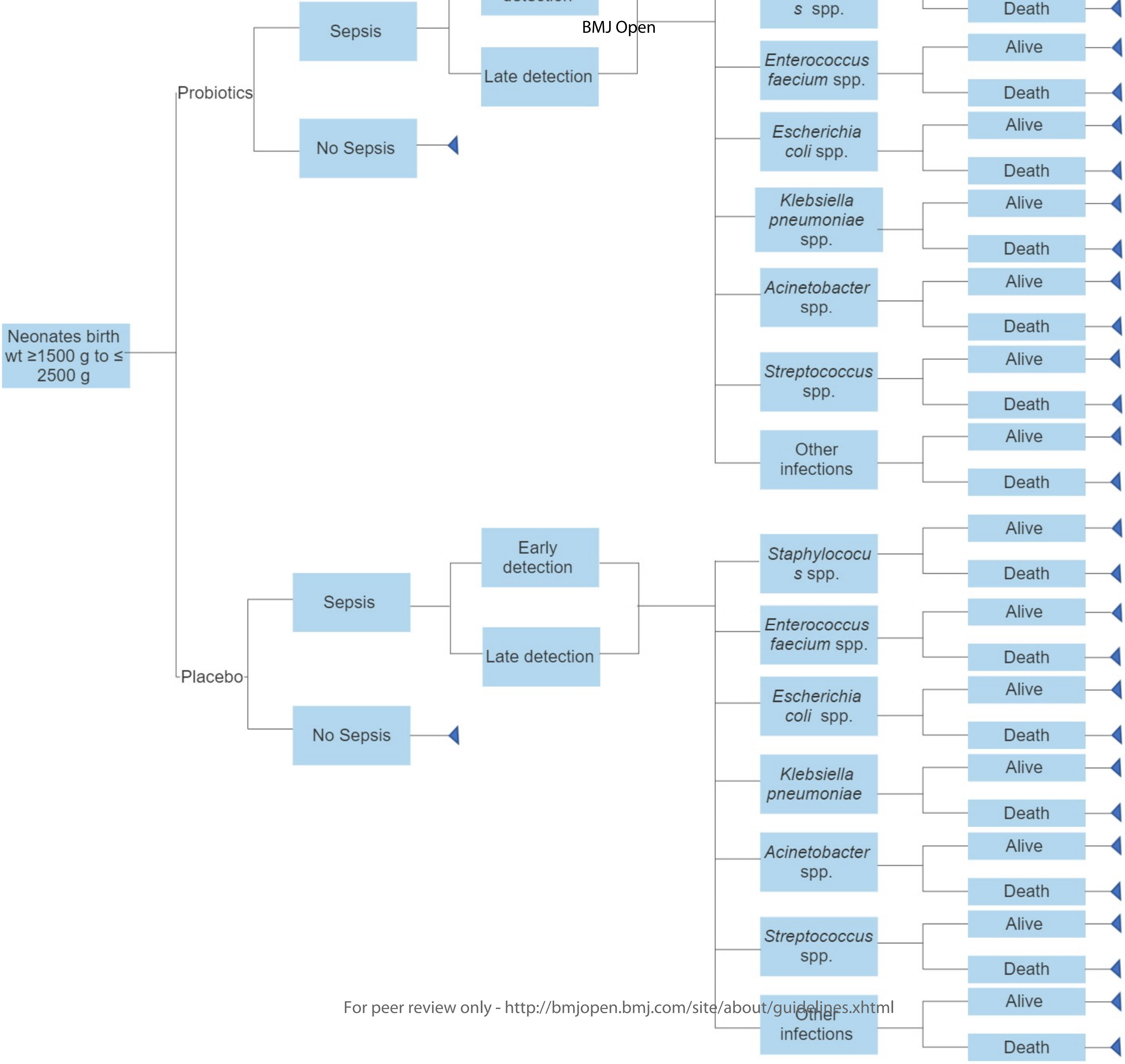

For peer review only - http://bmjopen.bmj.com/site/about/guidelines.xhtml

SPIRIT

STANDARD PROTOCOL ITEMS: RECOMMENDATIONS FOR INTERVENTIONAL TRIALS

SPIRIT 2013 Checklist: Recommended items to address in a clinical trial protocol and related documents*

Section/item	ItemNo	Description	Page number and line number
**Administrative information**			
Title	1	Descriptive title identifying the study design, population, interventions, and, if applicable, trial acronym	Page No. 1 Line No. 3-5
Trial registration	2a	Trial identifier and registry name. If not yet registered, name of intended registry	Page No. 15 Line No. 6-9
	2b	All items from the World Health Organization Trial Registration Data Set	Trial was registered in CTRI which is govern by WHO
Protocol version	3	Date and version identifier	Page No. 15 Line No. 6-9
Funding	4	Sources and types of financial, material, and other support	Page No. 16 Line No. 7-11
Roles and responsibilities	5a	Names, affiliations, and roles of protocol contributors	Page No. 15 Line No. 16-29<br>Page No. 16 Line No. 1-3
	5b	Name and contact information for the trial sponsor	Page No. 16 Line No. 7-11
	5c	Role of study sponsor and funders, if any, in study design; collection, management, analysis, and interpretation of data; writing of the report; and the decision to submit the report for publication, including whether they will have ultimate authority over any of these activities	No

For peer review only - http://bmjopen.bmj.com/site/about/guidelines.xhtml

	5d	Composition, roles, and responsibilities of the coordinating centre, steering committee, endpoint adjudication committee, data management team, and other individuals or groups overseeing the trial, if applicable (see Item 21a for data monitoring committee)	Described in the below given study protocol at Page No.3 Sinha AP, Gupta SS, Poluru R, Raut AV, Arora NK, Pandey RM, Sahu AR, Bethou A, Sazawal S, Parida S, Bavdekar A. Evaluating the efficacy of a multistrain probiotic supplementation for prevention of neonatal sepsis in 0–2-month-old low birth weight infants in India—the "ProSPoNS" Study protocol for a phase III, multicentric, randomized, double-blind, placebo-controlled trial. Trials. 2021 Dec;22(1):1-2.
**Introduction**			
Background and rationale	6a	Description of research question and justification for undertaking the trial, including summary of relevant studies (published and unpublished) examining benefits and harms for each intervention	Page No. 5<br>Line No. 4-23
	6b	Explanation for choice of comparators	Not applicable
Objectives	7	Specific objectives or hypotheses	Page No. 5<br>Line No. 24-25
Trial design	8	Description of trial design including type of trial (eg, parallel group, crossover, factorial, single group), allocation ratio, and framework (eg, superiority, equivalence, noninferiority, exploratory)	Page No. 8<br>Line No. 15-18
**Methods: Participants, interventions, and outcomes**			
Study setting	9	Description of study settings (eg, community clinic, academic hospital) and list of countries where data will be collected. Reference to where list of study sites can be obtained	Page No. 4<br>Line No. 2-12

For peer review only

Eligibility criteria	10	Inclusion and exclusion criteria for participants. If applicable, eligibility criteria for study centres and individuals who will perform the interventions (eg, surgeons, psychotherapists)	Described in the below given study protocol at Page No.5-6<br>Sinha AP, Gupta SS, Poluru R, Raut AV, Arora NK, Pandey RM, Sahu AR, Bethou A, Sazawal S, Parida S, Bavdekar A. Evaluating the efficacy of a multistrain probiotic supplementation for prevention of neonatal sepsis in 0–2-month-old low birth weight infants in India—the "ProSPoNS" Study protocol for a phase III, multicentric, randomized, double-blind, placebo-controlled trial. Trials. 2021 Dec;22(1):1-2.
Interventions	11a	Interventions for each group with sufficient detail to allow replication, including how and when they will be administered	Page No. 8<br>Line No. 15-17
	11b	Criteria for discontinuing or modifying allocated interventions for a given trial participant (eg, drug dose change in response to harms, participant request, or improving/worsening disease)	Not applicable
	11c	Strategies to improve adherence to intervention protocols, and any procedures for monitoring adherence (eg, drug tablet return, laboratory tests)	Page No. 8<br>Line No. 18-21
	11d	Relevant concomitant care and interventions that are permitted or prohibited during the trial	Page No. 8<br>Line No. 27-31
Outcomes	12	Primary, secondary, and other outcomes, including the specific measurement variable (eg, systolic blood pressure), analysis metric (eg, change from baseline, final value, time to event), method of aggregation (eg, median, proportion), and time point for each outcome. Explanation of the clinical relevance of chosen efficacy and harm outcomes is strongly recommended	Page No. 9<br>Line No. 2-23<br>Page No. 11-12<br>Table 2

Participant timeline	13	Time schedule of enrolment, interventions (including any run-ins and washouts), assessments, and visits for participants. A schematic diagram is highly recommended (see Figure)	Described in the below given study protocol at Page No.6 table. 1<br>Sinha AP, Gupta SS, Poluru R, Raut AV, Arora NK, Pandey RM, Sahu AR, Bethou A, Sazawal S, Parida S, Bavdekar A. Evaluating the efficacy of a multistrain probiotic supplementation for prevention of neonatal sepsis in 0–2-month-old low birth weight infants in India—the "ProSPoNS" Study protocol for a phase III, multicentric, randomized, double-blind, placebo-controlled trial. Trials. 2021 Dec;22(1):1-2.
Sample size	14	Estimated number of participants needed to achieve study objectives and how it was determined, including clinical and statistical assumptions supporting any sample size calculations	Page No. 10<br>Line No. 4-23
Recruitment	15	Strategies for achieving adequate participant enrolment to reach target sample size	Described in the below given study protocol at Page No.4<br>Sinha AP, Gupta SS, Poluru R, Raut AV, Arora NK, Pandey RM, Sahu AR, Bethou A, Sazawal S, Parida S, Bavdekar A. Evaluating the efficacy of a multistrain probiotic supplementation for prevention of neonatal sepsis in 0–2-month-old low birth weight infants in India—the "ProSPoNS" Study protocol for a phase III, multicentric, randomized, double-blind, placebo-controlled trial. Trials. 2021 Dec;22(1):1-2.
**Methods: Assignment of interventions (for controlled trials)**			
Allocation:			

Sequence generation	16a	Method of generating the allocation sequence (eg, computer-generated random numbers), and list of any factors for stratification. To reduce predictability of a random sequence, details of any planned restriction (eg, blocking) should be provided in a separate document that is unavailable to those who enrol participants or assign interventions	Page No. 8<br>Line No. 17-18
Allocation concealment mechanism	16b	Mechanism of implementing the allocation sequence (eg, central telephone; sequentially numbered, opaque, sealed envelopes), describing any steps to conceal the sequence until interventions are assigned	Described in the below given study protocol at Page No.7<br>Sinha AP, Gupta SS, Poluru R, Raut AV, Arora NK, Pandey RM, Sahu AR, Bethou A, Sazawal S, Parida S, Bavdekar A. Evaluating the efficacy of a multistrain probiotic supplementation for prevention of neonatal sepsis in 0–2-month-old low birth weight infants in India—the "ProSPoNS" Study protocol for a phase III, multicentric, randomized, double-blind, placebo-controlled trial. Trials. 2021 Dec;22(1):1-2.
Implementation	16c	Who will generate the allocation sequence, who will enrol participants, and who will assign participants to interventions	Described in the below given study protocol at Page No.4<br>Sinha AP, Gupta SS, Poluru R, Raut AV, Arora NK, Pandey RM, Sahu AR, Bethou A, Sazawal S, Parida S, Bavdekar A. Evaluating the efficacy of a multistrain probiotic supplementation for prevention of neonatal sepsis in 0–2-month-old low birth weight infants in India—the "ProSPoNS" Study protocol for a phase III, multicentric, randomized, double-blind, placebo-controlled trial. Trials. 2021 Dec;22(1):1-2.

Blinding (masking)	17a	Who will be blinded after assignment to interventions (eg, trial participants, care providers, outcome assessors, data analysts), and how	Described in the below given study protocol at Page No.7 Sinha AP, Gupta SS, Poluru R, Raut AV, Arora NK, Pandey RM, Sahu AR, Bethou A, Sazawal S, Parida S, Bavdekar A. Evaluating the efficacy of a multistrain probiotic supplementation for prevention of neonatal sepsis in 0–2-month-old low birth weight infants in India—the "ProSPoNS" Study protocol for a phase III, multicentric, randomized, double-blind, placebo-controlled trial. Trials. 2021 Dec;22(1):1-2.
	17b	If blinded, circumstances under which unblinding is permissible, and procedure for revealing a participant's allocated intervention during the trial	Not applicable
**Methods: Data collection, management, and analysis**			
Data collection methods	18a	Plans for assessment and collection of outcome, baseline, and other trial data, including any related processes to promote data quality (eg, duplicate measurements, training of assessors) and a description of study instruments (eg, questionnaires, laboratory tests) along with their reliability and validity, if known. Reference to where data collection forms can be found, if not in the protocol	Page No. 10<br>Line No. 25-35<br><br>Page No. 11<br>Line No. 1-6

For peer review only - http://bmjopen.bmj.com/site/about/guidelines.xhtml

	18b	Plans to promote participant retention and complete follow-up, including list of any outcome data to be collected for participants who discontinue or deviate from intervention protocols	Described in the below given study protocol at Page No.4 Sinha AP, Gupta SS, Poluru R, Raut AV, Arora NK, Pandey RM, Sahu AR, Bethou A, Sazawal S, Parida S, Bavdekar A. Evaluating the efficacy of a multistrain probiotic supplementation for prevention of neonatal sepsis in 0–2-month-old low birth weight infants in India—the "ProSPoNS" Study protocol for a phase III, multicentric, randomized, double-blind, placebo-controlled trial. Trials. 2021 Dec;22(1):1-2.
Data management	19	Plans for data entry, coding, security, and storage, including any related processes to promote data quality (eg, double data entry; range checks for data values). Reference to where details of data management procedures can be found, if not in the protocol	Described in the below given study protocol at Page No.8 Sinha AP, Gupta SS, Poluru R, Raut AV, Arora NK, Pandey RM, Sahu AR, Bethou A, Sazawal S, Parida S, Bavdekar A. Evaluating the efficacy of a multistrain probiotic supplementation for prevention of neonatal sepsis in 0–2-month-old low birth weight infants in India—the "ProSPoNS" Study protocol for a phase III, multicentric, randomized, double-blind, placebo-controlled trial. Trials. 2021 Dec;22(1):1-2.
Statistical methods	20a	Statistical methods for analysing primary and secondary outcomes. Reference to where other details of the statistical analysis plan can be found, if not in the protocol	Page No. 12<br>Line No. 2-6
	20b	Methods for any additional analyses (eg, subgroup and adjusted analyses)	Page No. 12<br>Line No. 9-14<br>Page No. 13<br>Line No. 1-12

For peer review only - http://bmjopen.bmj.com/site/about/guidelines.xhtml

	20c	Definition of analysis population relating to protocol non-adherence (eg, as randomised analysis), and any statistical methods to handle missing data (eg, multiple imputation)	Described in the below given study protocol at Page No.8 Sinha AP, Gupta SS, Poluru R, Raut AV, Arora NK, Pandey RM, Sahu AR, Bethou A, Sazawal S, Parida S, Bavdekar A. Evaluating the efficacy of a multistrain probiotic supplementation for prevention of neonatal sepsis in 0–2-month-old low birth weight infants in India—the "ProSPoNS" Study protocol for a phase III, multicentric, randomized, double-blind, placebo-controlled trial. Trials. 2021 Dec;22(1):1-2.
**Methods: Monitoring**			
Data monitoring	21a	Composition of data monitoring committee (DMC); summary of its role and reporting structure; statement of whether it is independent from the sponsor and competing interests; and reference to where further details about its charter can be found, if not in the protocol. Alternatively, an explanation of why a DMC is not needed	Described in the below given study protocol at Page No.3 Sinha AP, Gupta SS, Poluru R, Raut AV, Arora NK, Pandey RM, Sahu AR, Bethou A, Sazawal S, Parida S, Bavdekar A. Evaluating the efficacy of a multistrain probiotic supplementation for prevention of neonatal sepsis in 0–2-month-old low birth weight infants in India—the "ProSPoNS" Study protocol for a phase III, multicentric, randomized, double-blind, placebo-controlled trial. Trials. 2021 Dec;22(1):1-2.

	21b	Description of any interim analyses and stopping guidelines, including who will have access to these interim results and make the final decision to terminate the trial	Described in the below given study protocol at Page No.8 Sinha AP, Gupta SS, Poluru R, Raut AV, Arora NK, Pandey RM, Sahu AR, Bethou A, Sazawal S, Parida S, Bavdekar A. Evaluating the efficacy of a multistrain probiotic supplementation for prevention of neonatal sepsis in 0–2-month-old low birth weight infants in India—the "ProSPoNS" Study protocol for a phase III, multicentric, randomized, double-blind, placebo-controlled trial. Trials. 2021 Dec;22(1):1-2.
Harms	22	Plans for collecting, assessing, reporting, and managing solicited and spontaneously reported adverse events and other unintended effects of trial interventions or trial conduct	Page No. 8<br>Line No. 23-31
Auditing	23	Frequency and procedures for auditing trial conduct, if any, and whether the process will be independent from investigators and the sponsor	This being regulatory trial it is directly monitored by the DCGI
**Ethics and dissemination**			
Research ethics approval	24	Plans for seeking research ethics committee/institutional review board (REC/IRB) approval	Page No. 15<br>Line No. 10-14
Protocol amendments	25	Plans for communicating important protocol modifications (eg, changes to eligibility criteria, outcomes, analyses) to relevant parties (eg, investigators, REC/IRBs, trial participants, trial registries, journals, regulators)	We follow DCGI regulations

Consent or assent	26a	Who will obtain informed consent or assent from potential trial participants or authorised surrogates, and how (see Item 32)	Described in the below given study protocol at Page No.4 Sinha AP, Gupta SS, Poluru R, Raut AV, Arora NK, Pandey RM, Sahu AR, Bethou A, Sazawal S, Parida S, Bavdekar A. Evaluating the efficacy of a multistrain probiotic supplementation for prevention of neonatal sepsis in 0–2-month-old low birth weight infants in India—the "ProSPoNS" Study protocol for a phase III, multicentric, randomized, double-blind, placebo-controlled trial. Trials. 2021 Dec;22(1):1-2.
	26b	Additional consent provisions for collection and use of participant data and biological specimens in ancillary studies, if applicable	On 60th day of follow up additional informed consent was taken from the participants for Health economics study
Confidentiality	27	How personal information about potential and enrolled participants will be collected, shared, and maintained in order to protect confidentiality before, during, and after the trial	anonymize identifiable information
Declaration of interests	28	Financial and other competing interests for principal investigators for the overall trial and each study site	Page No. 16 Line No. 7-12
Access to data	29	Statement of who will have access to the final trial dataset, and disclosure of contractual agreements that limit such access for investigators	Page No. 16 Line No. 13
Ancillary and post-trial care	30	Provisions, if any, for ancillary and post-trial care, and for compensation to those who suffer harm from trial participation	As per GCP guidelines all these will be provided to participants on request of site Principal Investigator

For peer review only - http://bmjopen.bmj.com/site/about/guidelines.xhtml

Dissemination policy	31a	Plans for investigators and sponsor to communicate trial results to participants, healthcare professionals, the public, and other relevant groups (eg, via publication, reporting in results databases, or other data sharing arrangements), including any publication restrictions	Page No. 15 Line No. 12-13
	31b	Authorship eligibility guidelines and any intended use of professional writers	Page No. 15 Line No. 25-29 Page No.16 Line No.1-3
	31c	Plans, if any, for granting public access to the full protocol, participant-level dataset, and statistical code	Not yet planed
**Appendices**			
Informed consent materials	32	Model consent form and other related documentation given to participants and authorised surrogates	Uploaded as 'Supplemental Material' file
Biological specimens	33	Plans for collection, laboratory evaluation, and storage of biological specimens for genetic or molecular analysis in the current trial and for future use in ancillary studies, if applicable	As per existing SOP lab specific procedures

*It is strongly recommended that this checklist be read in conjunction with the SPIRIT 2013 Explanation & Elaboration for important clarification on the items. Amendments to the protocol should be tracked and dated. The SPIRIT checklist is copyrighted by the SPIRIT Group under the Creative Commons "Attribution-NonCommercial-NoDerivs 3.0 Unported" license.

For peer review only - http://bmjopen.bmj.com/site/about/guidelines.xhtml
